# An Artificial Neural Network Integrated Pipeline for Biomarker Discovery Using Alzheimer's Disease as a Case Study

**DOI:** 10.1016/j.csbj.2018.02.001

**Published:** 2018-02-21

**Authors:** Dimitrios Zafeiris, Sergio Rutella, Graham Roy Ball

**Affiliations:** John van Geest Cancer Research Centre, College of Science and Technology, Nottingham Trent University, United Kingdom

**Keywords:** ANN, artificial neural network, AD, Alzheimer's disease, MLP, multi-layer perceptron, APP, amyloid precursor protein, Aβ, beta amyloid, NFT, neurofibrillary tangles, Artificial neural network, Machine learning, Supervised learning, Network inference, Alzheimer's disease, Biomarker discovery

## Abstract

The field of machine learning has allowed researchers to generate and analyse vast amounts of data using a wide variety of methodologies. Artificial Neural Networks (ANN) are some of the most commonly used statistical models and have been successful in biomarker discovery studies in multiple disease types. This review seeks to explore and evaluate an integrated ANN pipeline for biomarker discovery and validation in Alzheimer's disease, the most common form of dementia worldwide with no proven cause and no available cure. The proposed pipeline consists of analysing public data with a categorical and continuous stepwise algorithm and further examination through network inference to predict gene interactions. This methodology can reliably generate novel markers and further examine known ones and can be used to guide future research in Alzheimer's disease.

## 1 Introduction

### 1.1 Machine Learning

One of the biggest challenges that has arisen as part of the recent advances in the field of bioinformatics, is the vast amount of data that is being generated at an ever-increasing pace [[1], [2], [3]]. Utilising techniques such as next generation RNA and DNA sequencing, researchers have been able to provide access to exceptionally precise information on entire genomes [4]. This massive volume of data has created a problem of complexity, making it impossible to interrogate the data with traditional methodologies and provide answers with the desired degree of accuracy.

Machine learning is an interdisciplinary field of bioinformatics that involves a data-driven class of algorithms that seek to find solutions to a given problem by studying patterns in datasets based on factors such as gene expression and clinical information across a multitude of cases. These approaches have been widely and successfully used in biology, particularly in biomarker discovery studies [5,6], due to the versatility and power afforded by them and has resulted in a wide variety of machine learning algorithms and methodologies. This review seeks to explore the potential of an Artificial Neural Network (ANN) based pipeline to discover, analyse and validate novel biomarkers in diverse diseases. For this purpose, Alzheimer's disease (AD) will be used since the cause of the condition is poorly understood and there is no widely available cure or treatment.

### 1.2 Supervised Learning

Supervised learning approaches, the mechanisms of which are further discussed in chapter 3, are widely applied and use source features to predict a target class [7]. The supervised approach allows the algorithm to train itself by detecting patterns in large data sets that are predictive of the target class, such as highlighting the variance at the genetic level between AD and cognitively normal individuals. We can also make use of previous studies and adjust the algorithm parameters so that it accounts for this information, which allows the power of this approach to increase over time and produce more accurate and robust results. One major advantage of supervised learning is that such approaches are tolerant of the highly complex, nonlinear and noisy data that are often found in biological systems.

### 1.3 Artificial Neural Networks

ANNs are statistical models that emulate the function of a network of human neurons, for the purpose of encapsulating information in order to analyse large, complex datasets. The learning process is based on the mathematical interconnections between the processing elements that constitute the network architecture [8]. This allows them to classify cases based on data by assigning a numerical weight value to each input and adjust them as they sample the data, effectively learning the optimal solution. The main advantages of using ANNs include their high fault and failure tolerance, scalability and consistent generalisation ability, which allows them to predict or classify well for new, fuzzy and unlearned data [8,9]. This makes the ideal for biomarker studies which resulted in their use in generating panels of biomarkers that can be used as predictors in conjunction with each to aid prognosis in diseases such as breast cancer [10].

ANN architecture is based on the perceptron, coined by Rosenblatt in 1958, which is composed of a single artificial processing neuron with an activation threshold, adjustable weights and bias, but only usable for the classification of linearly separable patterns, as learning is achieved when an error occurs during testing. This is rarely the case with complex conditions such as AD, cancer or diabetes, as patients rarely fall in a standard distribution and the variance between them is potentially significant. Typically, ANNs make use of a Multi-Layer Perceptron (MLP) which is made up of multiple perceptrons arranged in layers of three or more, consisting of input, hidden and output layers, which consider predictor variables, perform feature detection through an activation function and output the results of the algorithm respectively.

Alternative ANN architectures include Recurrent Neural Networks, Radial Basis Function, Kohonen's self-organizing maps and Adaptive Resonance Theory but the focus of this review will be on the MLP.

ANNs have seen widespread success in predicting and classifying data in multiple cancer subtypes such as early detection [11], prediction of long term survival [12] and biomarker discovery in breast cancer [10,13], classifying colorectal cancer tissues [14] and discriminating between benign and malignant endothelial lesions [15]. Thus, we are confident that they will see similar success in AD.

The main ANN disadvantage is their liability to overfit when the parameters have not been optimised and often receive criticism for their “black box” approach that allows for little interpretation of the results and process.

### 1.4 Alzheimer's Disease

**Fig. 1:**
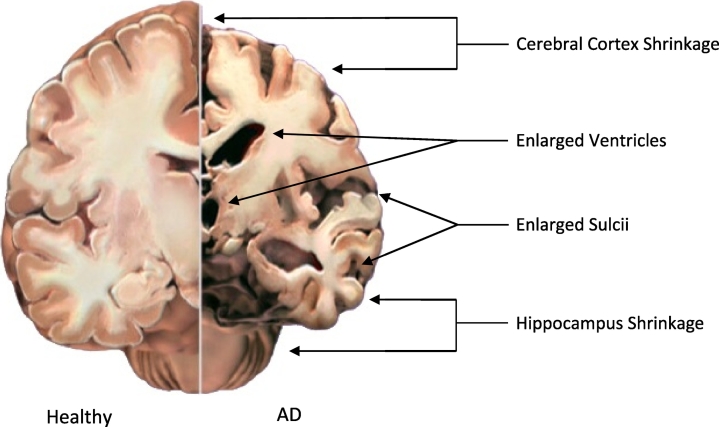
Physiological differences between a healthy and AD brain section, demonstrating white matter shrinkage in the hippocampus and cerebral cortex.

Source: www.alz.org.

Alzheimer's disease is recognised as the most common form of dementia worldwide. This chronic neurodegenerative disease usually starts slowly, with the common early symptom being difficulty to remember short-term events and progressively getting worse, with severe degeneration of multiple brain regions including the hippocampus, entorhinal cortex, neocortex, nucleus basalis, locus coeruleus and raphe nuclei (Fig. 1), leading to disruption in mental functions such as comprehension, judgement, language and calculation. Moreover, due to slow progression that characterises the disease as well as common misconceptions, it is common for patients and their families to assume that this degeneration is a normal part of ageing, thus delaying early prognosis. It is crucial to emphasise that AD is the abnormal degeneration of mental faculties and while age is indeed the biggest risk factor, it is far from the only one.

In addition to the enormous emotional cost the disease exerts on patients and their families, it has become a major public concern due to the high healthcare costs which, in combination with the overall rise in the elderly population has classified AD as a priority condition [16]. According to the World Health organisation, in 2015 there were over 40 million people with dementia in the US, 15 million of which suffered from Alzheimer's disease. Healthcare costs have spiralled to over USD 900 billion, whereas in Europe the costs have risen to nearly 250 billion euros, a rise of almost 40% from 2008. Moreover, it is projected that by 2050, 22% of the world's population will be over the age of 60, and therefore at increased risk, with patients in third world countries accounting for 80% of the total.

### 1.5 Theories and Treatments

Compounding the social and economic challenges presented by the disease is the fact that its root causes are unknown and there is no cure or effective treatment. While there is a small percentage of the population, 1–5% of all cases, that suffer from early onset AD, which is caused by mutations in the amyloid precursor protein gene (APP) and the two presenilin genes PSEN-1 and PSEN-2, the cause for the majority of late onset Alzheimer's cases is still unknown. In the last decade, clinically approved drugs for AD such as Cholinesterase inhibitors like Donepezil, Galantamine and Rivastigmine as well as *N*-methyl-d-aspartate antagonist Memantine [17] have not been able to make significant progress with the disorder.

Cholinesterase inhibitors, which target the cholinergic systems in the basal forebrain, where developed based on the theory that the loss of acetylcholine neurons during the early development of the disease inhibit the synthesis and degradation of acetylcholine, one of the major neurotransmitters in the brain. Therapy was targeted at patients with mild, moderate and severe AD but improvement of cognitive functions was noticeably better in patients that started treatment early [18]. *N*-methyl-d-aspartate antagonist on the other hand, is an uncompetitive moderate affinity antagonist, targeted at moderate to severe AD cases, with the purpose of protecting neurons from excitotoxicity. Other forms of therapy have focused on combinations of these drugs and treatment of the behavioural and psychological symptoms of the disease.

More recently, therapeutic approaches have been based on the amyloid hypothesis, attempting to slow, stop and reverse the development of amyloid plaques by inhibiting production of beta amyloid, as well as the hyperphosphorylation and deposition of tau protein. Finally, further research has been focusing on the effects of oxidative damage and chronic inflammation in the brain to determine their effects in the development and progression of AD. It is evident by the variety of approaches as well as the failure of most forms of therapy to reverse or even significantly slow the disease progression, that a deeper understanding of the pathogenesis of AD is urgently needed to effectively combat it.

### 1.6 Physiology of Alzheimer's Disease

**Fig. 2:**
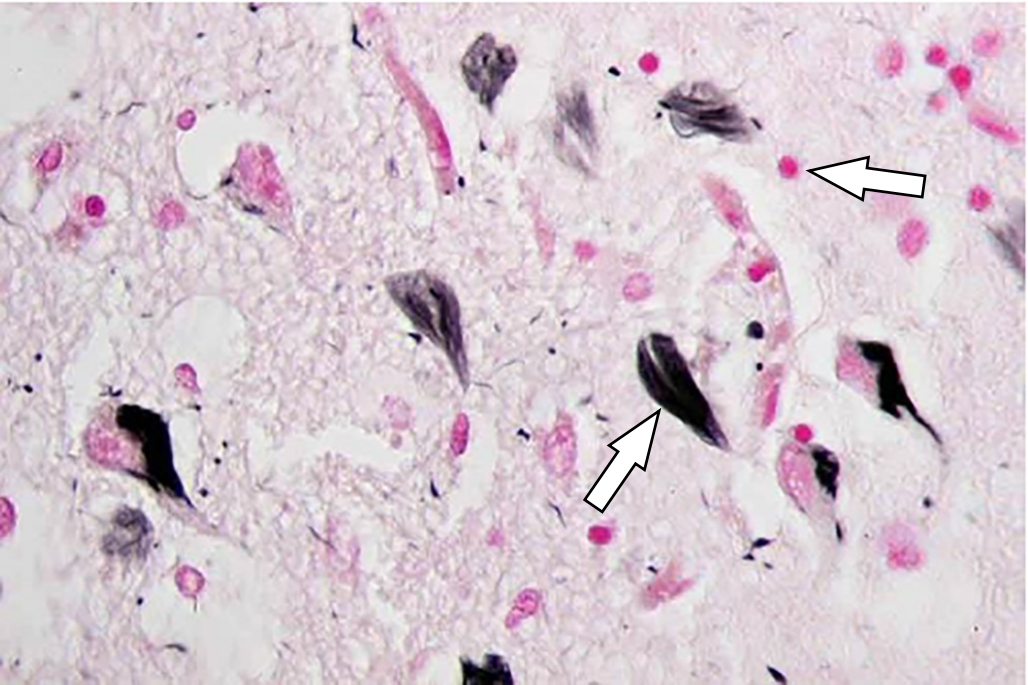
Amyloid plaques (pink) and neurofibrillary tangles (black) in Alzheimer's disease brain tissue.

Source: www.alzheimers.org.uk.

Historically, identification of AD could only be performed post mortem upon examination of the brain tissue. As a result, the physiological hallmarks of AD have been widely considered to be the presence of amyloid plaques, extracellular deposits of insoluble beta-amyloid (Aβ) in the parenchyma of the brain as well as neurofibrillary tangles (NFT), intracellular deposits of hyper-phosphorylated tau protein which fill the neuron and take its shape, preventing it from functioning correctly (Fig. 2).

Amyloid plaques consist of a solid core of defective Aβ and are surrounded by degenerate axons and dendrites, activated microglia and astrocytes. This defective protein is a result of the cleaving of the amyloid precursor protein (APP) by secretases beta (β) and gamma (γ). The location APP is cleaved by γ-secretase determines whether Aβ will be the long or short form. The short form is the most common (~90%) but the long form is found as often as 40% in the brains of AD patients [19], and while small amounts can be cleared easily, the high rate of production leads to the system being unable to keep up. Moreover, soluble forms of the protein have been shown to be neurotoxic and synaptotoxic [20].

Neurofibrillary tangles are a result of the hyperphosphorylation of tau, a microtubule associated protein (MAP) whose role is to bind to tubulin and stabilise the structure of neurons to maintain their function. When hyperphosphorylated due to excessive amounts of phosphate ions, it changes from its normal soluble form to oligomeric and fibrillized forms, does not bind to tubulin, inhibits microtubule structure and assembly and has been shown to have a neurotoxic effect [21].

### 1.7 The Amyloid Cascade Hypothesis

**Fig. 3:**
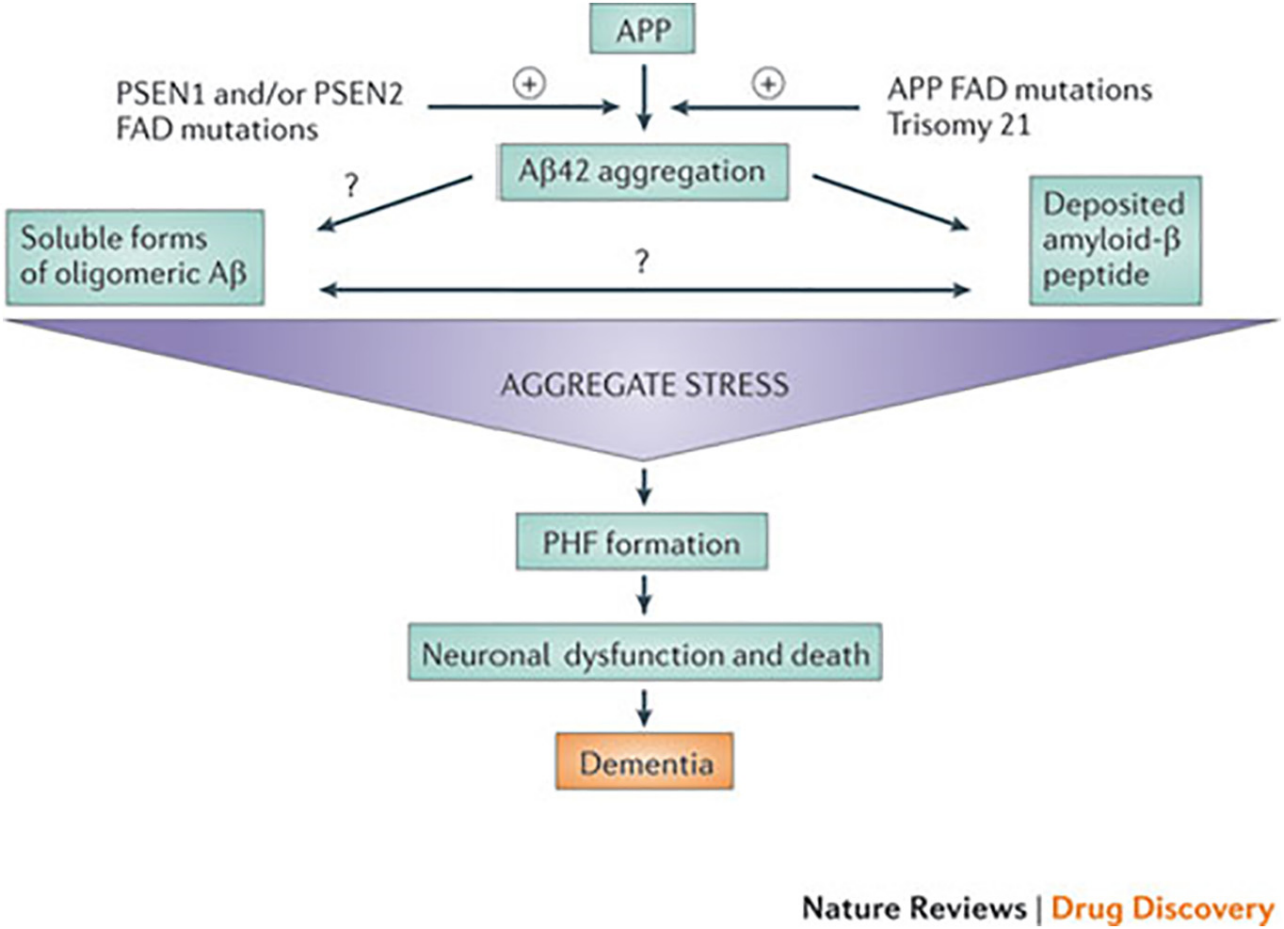
Diagram of the amyloid cascade hypothesis showing the theorised links between the aggregation of Aβ to cell death and dementia.

Source: Karran et al. [25].

The leading theory for the cause of Alzheimer's disease is the amyloid cascade hypothesis, first proposed in 1992 and its influence on AD research cannot be understated. The hypothesis posits that mutations in the APP and presenilin genes PSEN1 and PSEN2 leads to the deposition of Αβ in the brain which subsequently leads to the formation of NFTs, cell death and dementia. Experiments in animal models have shown that chemically or damage induced lesions lead to an increase in APP levels and accelerate the development of AD [22,23]. Unfortunately, all approaches based on the amyloid cascade have failed at Phase III clinical trials - tramiprosate, tarenflurbil and semagacestat - and research has not been able to conclusively link the build-up of Aβ to the formation of NFTs (Fig. 3) [24].

While it has been made clear that the amyloid cascade hypothesis is not enough to sufficiently explain the development of AD or aid in its detection and consequently, is currently under heavy scrutiny, it is also not possible to accept the null hypothesis, as autosomal dominant mutations in the aforementioned APP, PSEN1 and PSEN2 genes along with the apolipoprotein E4 (APOE4) allele have been proven to be the key components in familial, or early onset, Alzheimer's disease. Instead, the amyloid cascade hypothesis has to be modified to account for the rate of Aβ deposition and clearance, the connection with the development of NFTs and the effect of inflammation in the development of AD. Karran et al. [25] have attempted to update the hypothesis for use in therapeutics by presenting four distinct scenarios describing the role of Aβ in AD. These scenarios are:

1. Aβ could trigger development of the disease and further accumulation has little to no effect
2. development starts once Αβ reaches a certain, as yet unknown, threshold
3. Aβ is a key driver of AD and its continued deposition accelerates the effect
4. Aβ is irrelevant and the presence of plaques and increased levels of Aβ are a side effect of a different cause.

It should be noted that a major limitation of this hypothesis is that it fails to account for AD patients with little to no AD pathology [26] and thus amyloid plaques as identified by PET scan. In recent years, mice studies have shown that Aβ deposition is a potential driver for tau hyperphosphorylation, fixing one the major limitations of the amyloid hypothesis. Crossing APP transgenic mice with tau knockout mice, resulted in offspring with significantly fewer behavioural deficits [27] while other studies have shown that soluble oligomers of Aβ can lead to alterations in tau, potentially cascading to AD [28] although the mechanisms are still unclear. Strooper and Karran [29] attempted to provide alternatives including proteostatic stress during the biochemical phase when Aβ aggregates at an abnormally fast pace, defections in the amyloid and tau clearance mechanisms and a decrease in synaptic plasticity. As Selkoe and Hardy [27] suggest, the amyloid hypothesis, for all it limitations, is essential for therapeutics due to the fact that the complexity of the disease increases drastically after initiation due to the rise in complexity of downstream pathogenic processes, the most likely point of the disease where treatment will be at its most successful.

### 1.8 Inflammation in Alzheimer's Disease

**Fig. 4:**
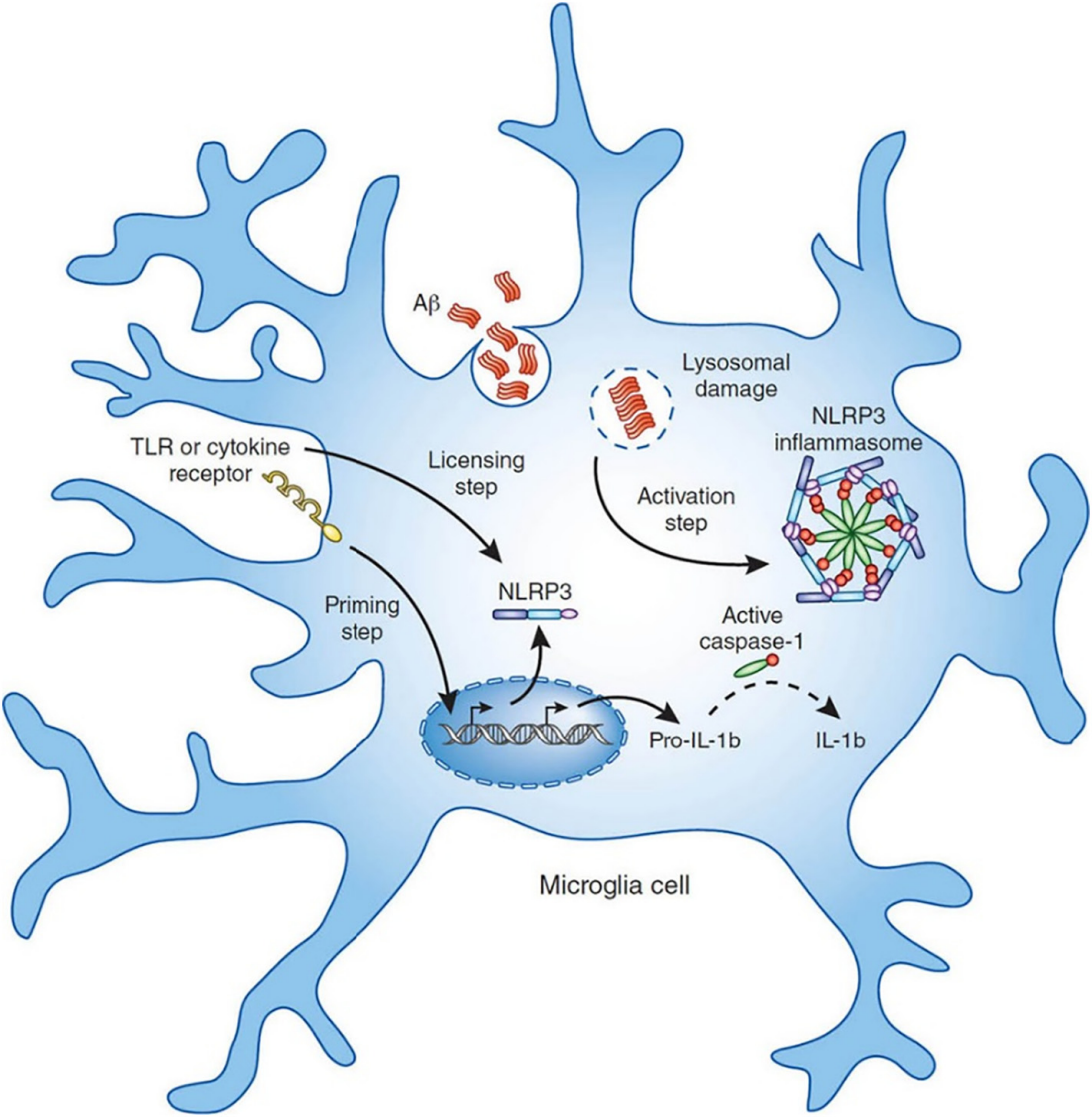
Microglial cell diagram showing the formation of the NLRP3 inflammasome and cytokine cascade as a result of Aβ detection.

Source: Heneka et al. [32].

Recent research has also been focused on investigating the role of inflammation in AD in an attempt to explain the development of the disease. The inflammation hypothesis posits that deposition of Aβ causes chronic activation of the immune system and disrupts microglial clearance functions. Microglia are immune cells located in the parenchyma of the brain, making up 20% of the total glial population. Their functions include phagocytosis, induction of inflammation, and antigen presentation to lymphocytes [30]. However, their roles also include clearance of extracellular deposits of Aβ, and microglial receptors TLR2, TLR4, TLR6 and co-receptors CD36, CD14 and CD47 activated upon detection of the protein. These receptors can also sense pathogen-associated molecular patterns such as bacterial lipopolysaccharides and viral surface proteins and thus are instrumental for mediating the immune response. Certain bacteria have similar surface amyloids, such as curli fibers, which resemble Aβ aggregates and thus activate toll-like receptors (TLR) and CD36, which in turn triggers the formation of a TLR4-TLR6 heterodimer and results in signalling activation via the transcription factor NF-κB. This leads to a cytokine cascade which further attracts immune cells to the site of the perceived infection (Fig. 4).

Moreover, certain cytokines such as IL-1β, damage the synaptic plasticity by disrupting the formation of dendritic spines, with high cytokine expression being able to disrupt normal hippocampus function. This lead to the hypothesis that chronic activation of the immune systems leads to chronic inflammation and microglial cell death, resulting in increased proliferation and accelerated senescence.

## 2 Artificial Neural Networks and Systems Biology

### 2.1 Artificial Neural Networks

**Fig. 5:**
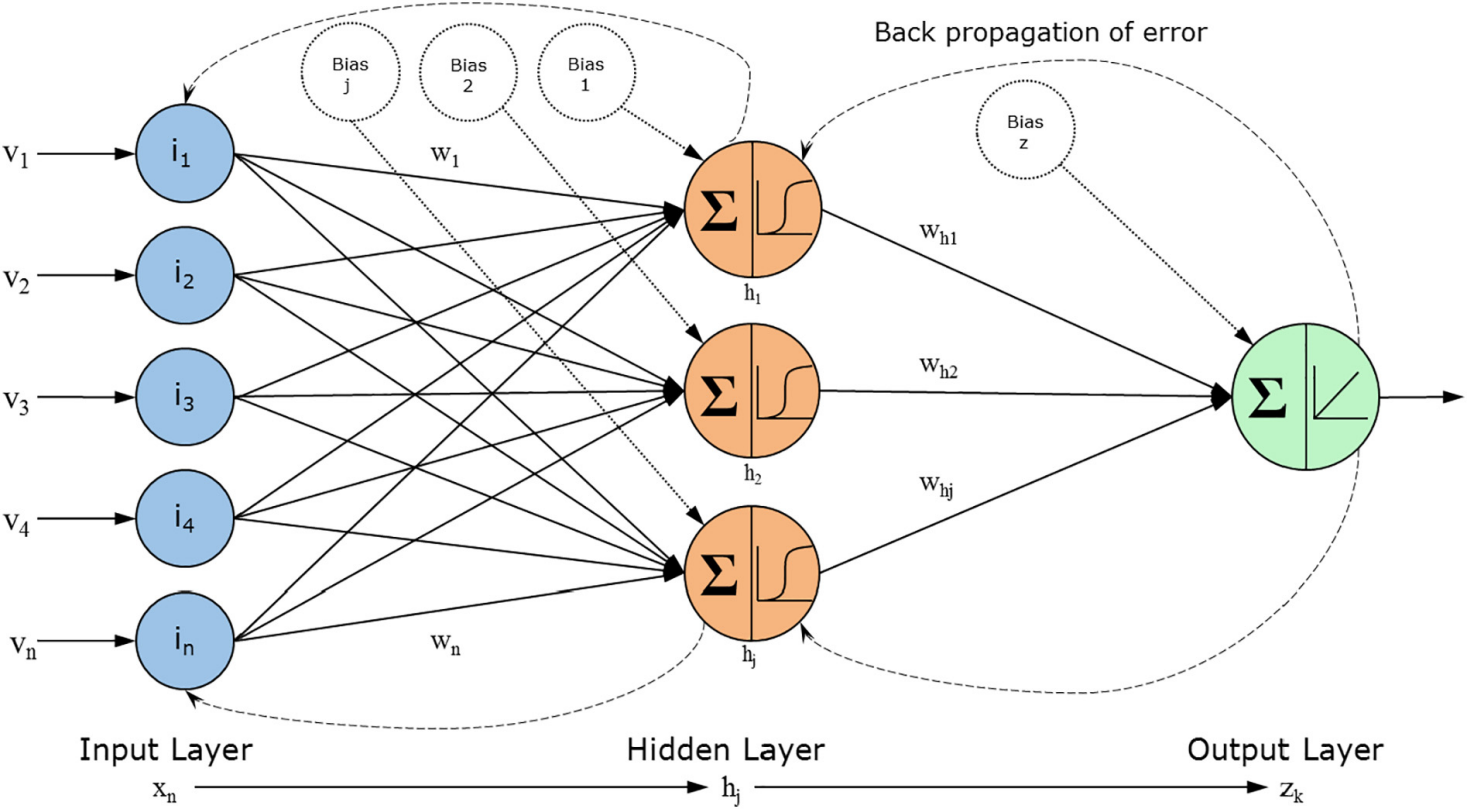
Workflow diagram of the artificial neural network algorithm developed by Lancashire et al. [31] used for this project. The parameters for the hidden and output layer nodes are in their paper.

As explained previously, ANNs are a form of machine learning, statistical models emulating the function of a neuron, able to identify patterns and linearly separate them by assigning a numerical weight value to each input and adjust them as they sample the data, effectively learning the optimal solution. They can make use of parallel processing in order to predict solutions to complex and non-linear data (Fig. 5) [31].

The ANN used for this project is a Multi-Layer Perceptron (MLP) with a back-propagation (BP) algorithm. It is organised in several layers, each with a number of mathematical processing elements depending on the complexity of the problem and the BP algorithm is responsible for feeding the error back through the model, allowing it to adjust the training weights accordingly and stop early if no gains can be made.

### 2.2 Stepwise Analysis

The stepwise ANN approach developed by Lancashire [33] allows for the identification of a gene or set of genes with the best predictive performance to classify samples based on a certain question by data mining the complete transcriptome. The ANN model functions by modifying the network weights and subsequently adding variables in an iterative manner to find a model with the lowest predictive error. The architecture consists of a single hidden layer, feed forward MLP with a variable number of hidden nodes and a sigmoidal transfer function, using a back-propagation algorithm incorporating supervised learning for updating the network weights. A Monte Carlo Cross Validation (MCCV) strategy was applied to produce a more generalized model with an improved predictive ability for unseen or future cases. The MCCV randomly divides the samples into training, test and validation subsets in 60:20:20 proportion for 50 iterations to provide the most consistent models. The parameters selected for this series of tests are 1 step, 10 loops with a momentum of 0.5, learning rate of 0.1 and threshold of 0.01 [34]. These parameters have been thoroughly tested and successfully used in other studies [10]. The dataset used for this experiment is [dataset] E-GEOD-48350 [35].

The dataset is publicly available and has been accessed using ArrayExpress [36] as well as the Gene Expression Omnibus (GEO) [37]. It was selected based on the following parameters to ensure high quality results:

• Human samples only
• Patient size of >80
• Genes in array >40,000
• A minimum of four brain region samples
• Healthy controls between 33% and 66% of the dataset
• Recent Publication
• Raw data in the form of CEL files available.

The methodology flowchart is included in the Supplemental Fig. 1. The outcome of the stepwise analysis is a list of genes, ordered from the most to least likely to explain the variance in the population based on AD status.

### 2.3 Categorical and Continuous

It is worth noting that two distinct versions of the algorithm were used – categorical and continuous. The categorical version seeks to interrogate the dataset using two predictors 0 and 1 for two distinct possibilities. This is based on known clinical information and a multitude of questions were considered. These questions include examining the differences between a healthy and an AD brain based on the overall gene expression as well as the differences between different regions in the brain, most notably the hippocampus. The continuous version of the algorithm allows us to consider every gene as its own independent predictor. This was used to examine the currently accepted biomarkers for AD [38,39] APP (amyloid beta precursor protein), MAPT (microtubule associated protein tau) and APOE (apolipoprotein E) and compare them to biomarkers discovered by the categorical algorithm.

### 2.4 Network Inference

The results obtained from the stepwise ANN approach were further analysed with an interaction algorithm developed by Lemetre et al. [34] to perform network inference. The interaction algorithm allows for the iterative quantification of the influence that multiple genes might have on the expression level of a single gene, until all the genes within the data have been quantified this way, using the same parameter values as those utilized for the ANN stepwise algorithm [34]. This allows for the determination of the central role of the most influential genes selected by the stepwise ANN within a system. The interaction algorithm predicts a single probe and assigns a weighted score which is directly proportional to the intensity of linkage between itself and the expression values of all other gene probes [35], while the intensity and directionality of the interaction between a source and target are determined based on the sum of the weights from an input to an output. The association between gene pairs can be bi- or unidirectional and be either stimulatory or inhibitory. This process was repeated until all gene probes were used as an output iteratively and a large matrix of interaction scores was generated by averaging values across 10 iterations. The results were visualised using Cytoscape. The methodology, proposed by Tong et al. [40], is a novel ANN designed to infer directed gene-gene interactions in a pairwise manner, allowing the user to observe how changes in a given genes leads to changes in other genes and the network as a whole. The flowchart is included in the Supplemental Fig. 2.

### 2.5 Interaction Matrix

One of the greatest problems encountered during the previous approach when they are used to predict a single best marker is the fact that the selection process is stochastic; there is a random probability element and while the results can be statistically significant, it makes the process imprecise. To counter that effect and increase the power of this method, the top 500 genes selected by the stepwise process were split into 5 datasets of 100 genes each and combined into 16 sets of 200 genes each for network inference. This specific number was selected as the stepwise algorithm performance started to plateau after the first 400 genes indicating that the differentiation between the given conditions – AD and healthy – was decreasing. Once the network inference was completed, the data was consolidated and the top 1000 strongest interactions were selected and visualised with Cytoscape.

**Fig. 6:**
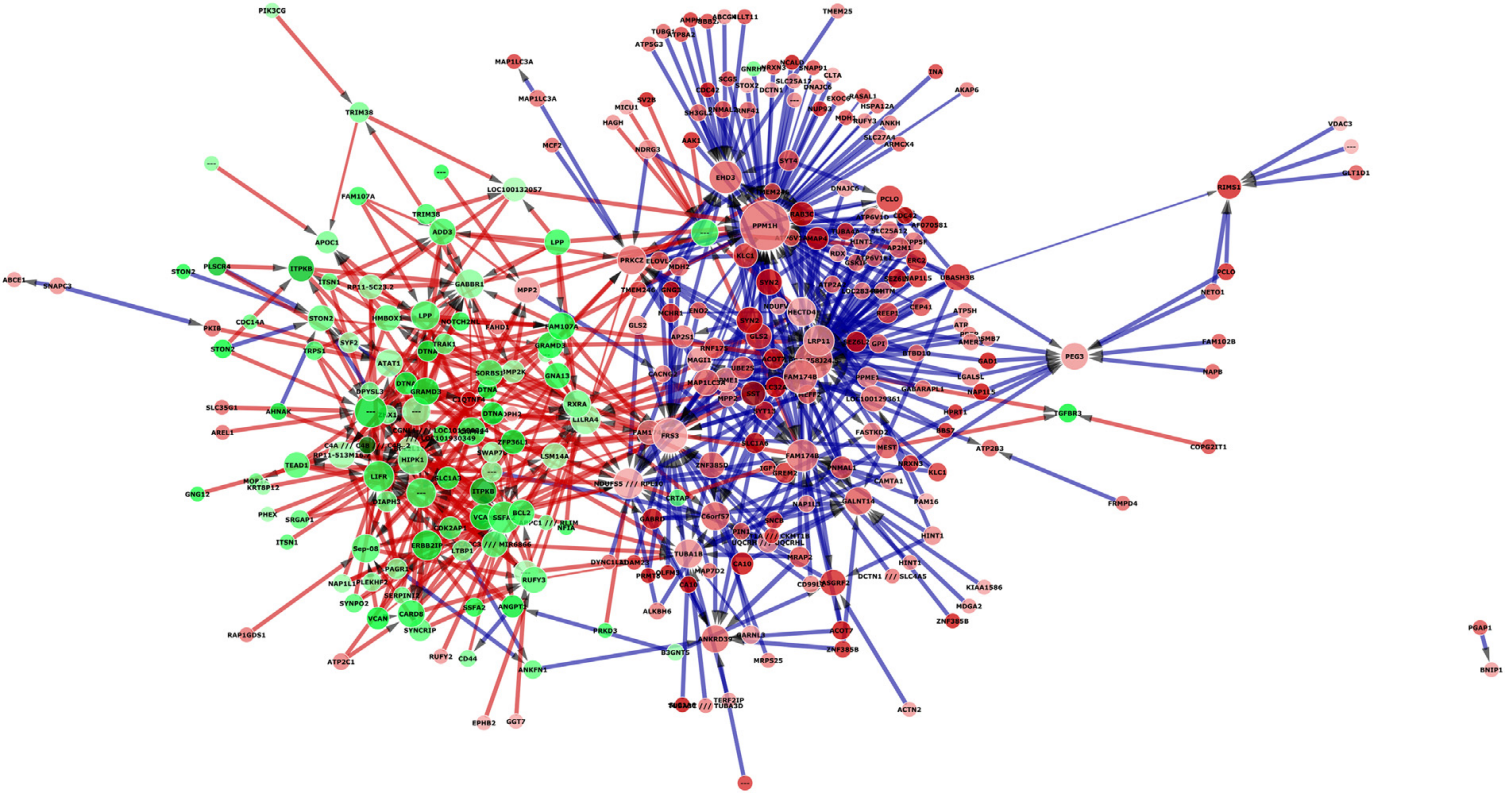
Force directed interactome encompassing 500 gene probes and 1000 predicted interactions of the hippocampus in the E-GEOD-48350 AD cohort. Red edges indicate and inhibitory effect, whereas blue edges indicate promotion. Edge thickness is directly proportional to the strength of the interaction. Green nodes are upregulated genes while red ones are downregulated. The intensity of the colour is directly proportional to the degree of up- or downregulation.

The reasoning behind developing this technique is that the normal single marker approach only focuses on a small subset (~0.1%) of the genes actively influencing a given condition. Moreover, by only selecting the 100 strongest interactions, it is guaranteed that in the resulting network, the biggest hubs, hence the most like drivers of the disease and targets for therapy, will be kept to a minimum and will be biased towards the most differentiated genes as seen in Fig. 6. It is important to note however, that for a highly focused system such as studying a specific subset of genes in a subset of a disease, such as proliferation markers in untreated breast cancer patients, the very nature of the data would result in a network where all the hubs are equally important. Thus, in such cases, identifying key markers and drivers using the strongest interactions is still the superior choice.

**Fig. 7:**
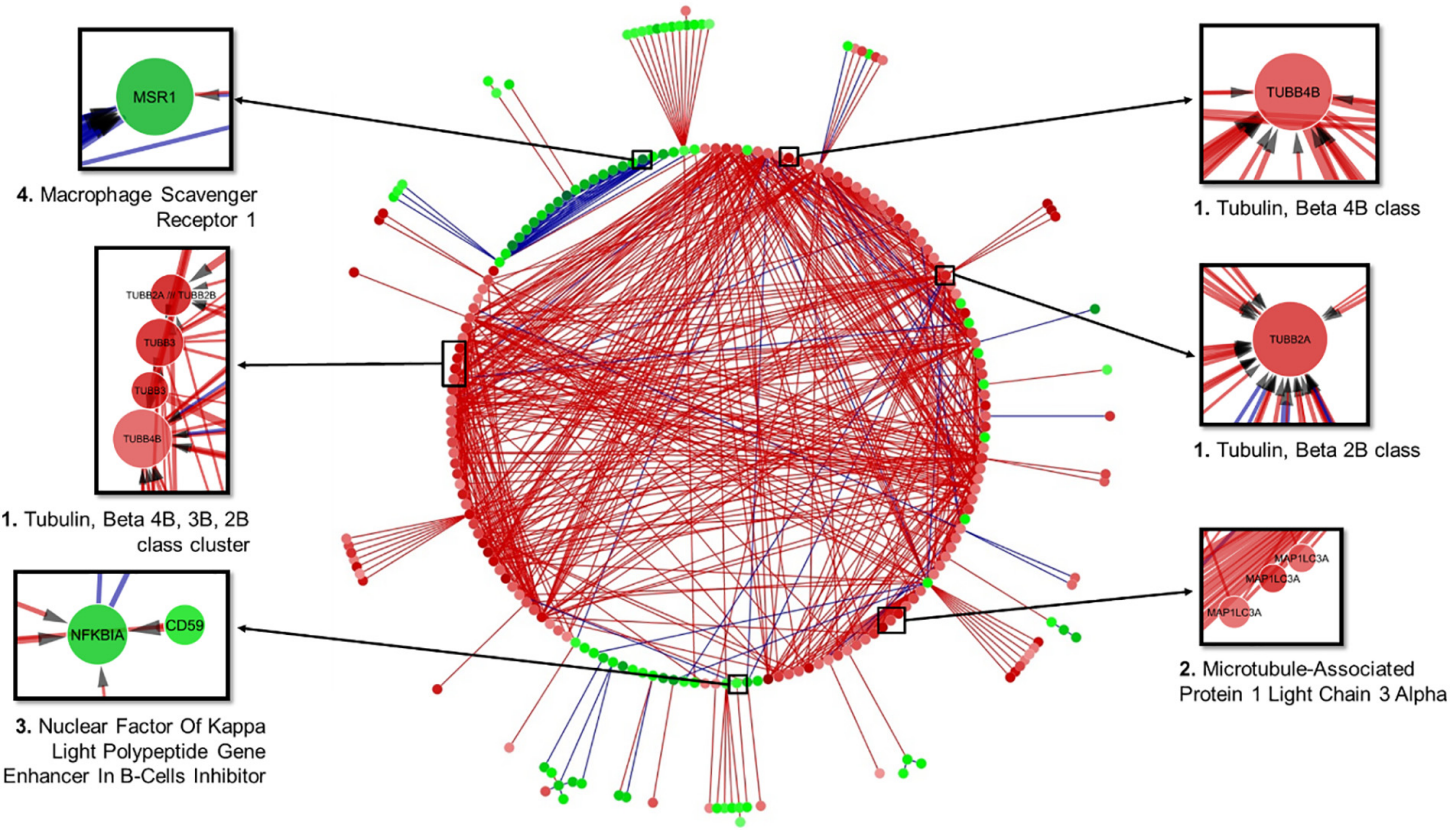
Alternative circular layout interactome of the 1000 strongest interactions between 500 genes in AD independent of the brain region in the E-GEOD-48350 dataset. Based on the overall expression of all brain regions. Novel targets identified. Red edges indicate and inhibitory effect, whereas blue edges indicate promotion. Edge thickness is directly proportional to the strength of the interaction. Green nodes are upregulated genes while red ones are downregulated. The intensity of the colour is directly proportional to the degree of up- or downregulation.

As seen in Fig. 6, upon separating the data to only include gene expression data exclusively from the hippocampus from AD patients only, selected as it is the area most strongly affected in AD, a rarely seen duality presents itself. In most complex diseases such as cancer, the dysregulation that is represented in such interactomes is a direct result of the mechanisms of the disease. Successful cancers can highjack the body's immune response, avoid detection and proliferate uncontrollably. This in turn, leads to the body mounting a very strong response by attempting to upregulate anti-tumour factors and suppress proliferation factors among others in order to prevent the abnormal cells from disrupting the function of crucial organs [10]. Diabetes is similarly represented, as due to chronically high sugar levels the function of the organs affected get significantly damaged [41]. This leads to interactomes that are either mostly up- or down-regulated.

However, irrespective of the cause, non-familial AD is a direct result of the failure to regenerate damaged cells and clean away debris over a long period of time. Moreover, the isolated nature of the brain, the increased regulation of substances that can cross the blood brain barrier and most importantly the brain's plasticity, are crucial defence factors other organs lack. Plasticity is especially important as the brain can tolerate extensive damage before showing significant dysregulation, which is why AD is so hard to identify early [42]. As a result, the interactomes of affected regions show both up- and downregulation as it is possible to observe both suppression factors that could potentially be the direct cause of the disease and healing factors that are attempting to restore balance, as the mechanisms for it are still present and functional. In fact, dysregulation in the mechanisms involved in immune response and debris clearance could be used as predictors for early prognosis of AD as they are still functional, but increasingly ineffective.

This duality in the interactome however, reveals an interesting pattern within the data. Based on a fold change analysis of the original microarray data for AD in E-GEOD-48350, the genes that are overexpressed are downregulated overall. Conversely, underexpressed genes are predicted to be mostly downregulated. It is a fact that the hippocampus is the most dysregulated brain region in AD, so this is possible proof that the system is attempting to restore balance by suppressing the high expression of factors such as HIPK1 [43], a kinase which plays an important role in senescence, ITPKB, a kinase that regulates inositol polyphosphates or BCL2, a protein phosphatase which is a crucial apoptosis factor. In short, the system is attempting to decrease the effect of genes involved in cell death.

The factors that are underexpressed on the other hand, appear to be upregulated and significantly more dysregulated, with an overall larger number and stronger individual interactions. The largest hub is PPM1H, another protein phosphatase which dephosphorylates CDKN1B, a CD kinase inhibitor involved in diseases such as Type IV Multiple Endocrine Neoplasia and familial Primary Hyperparathyroidism. Another such gene is FRS3, a fibroblast growth factor receptor substrate which is involved in regulation of RAS signalling.

While these genes and others like them seem to indicate that there is a significant effort to re-establish homeostasis, of further interest are the genes that do not fall inside these clearly defined categories. These genes include multiple tubulins such as TUBA1B and TUBB2A which are underexpressed but being simultaneously up- and downregulated, TGFBR3 which encodes for the transforming growth factor beta, type III receptor and plays a crucial role in cell adhesion and is associated with diseases such as familial cerebral saccular aneurysm. TGFB itself activates transcription factors of the SMAD family, which in turn, regulates gene expression. ATP2C1 is an ATPase which catalyses the hydrolysis of ATP and is underexpressed while still attempting to downregulate CARD8. CARD8 itself is caspase recruitment domain containing family of proteins and is involved in pathways negatively regulating the activation of NFKB, which as explained during the introduction, has a key role in the theory of neuroinflammation, and is quite likely an attempt to slow down or stop the chronic immune response leading to said neuroinflammation. Other irregularities include MAP1LC3A and MPP2 explained earlier and CD44, a cell-surface glycoprotein involved in cell-cell interactions, cell adhesion and migration and interacts with, among other things, matrix metalloproteinases (MMPs). MMPs, and MMP-9 in particular have long been suspected in playing a key role during AD and have been shown neuroprotective capabilities [44]. Finally, one of the most highly underexpressed and downregulated genes is C1QTNF4, a complement-C1q tumour necrosis factor-related protein whose role is not clearly defined but has been suspected of acting like a pro-inflammatory cytokine, leading to the activation of NFKB and upregulate production of IL6.

**Fig. 8:**
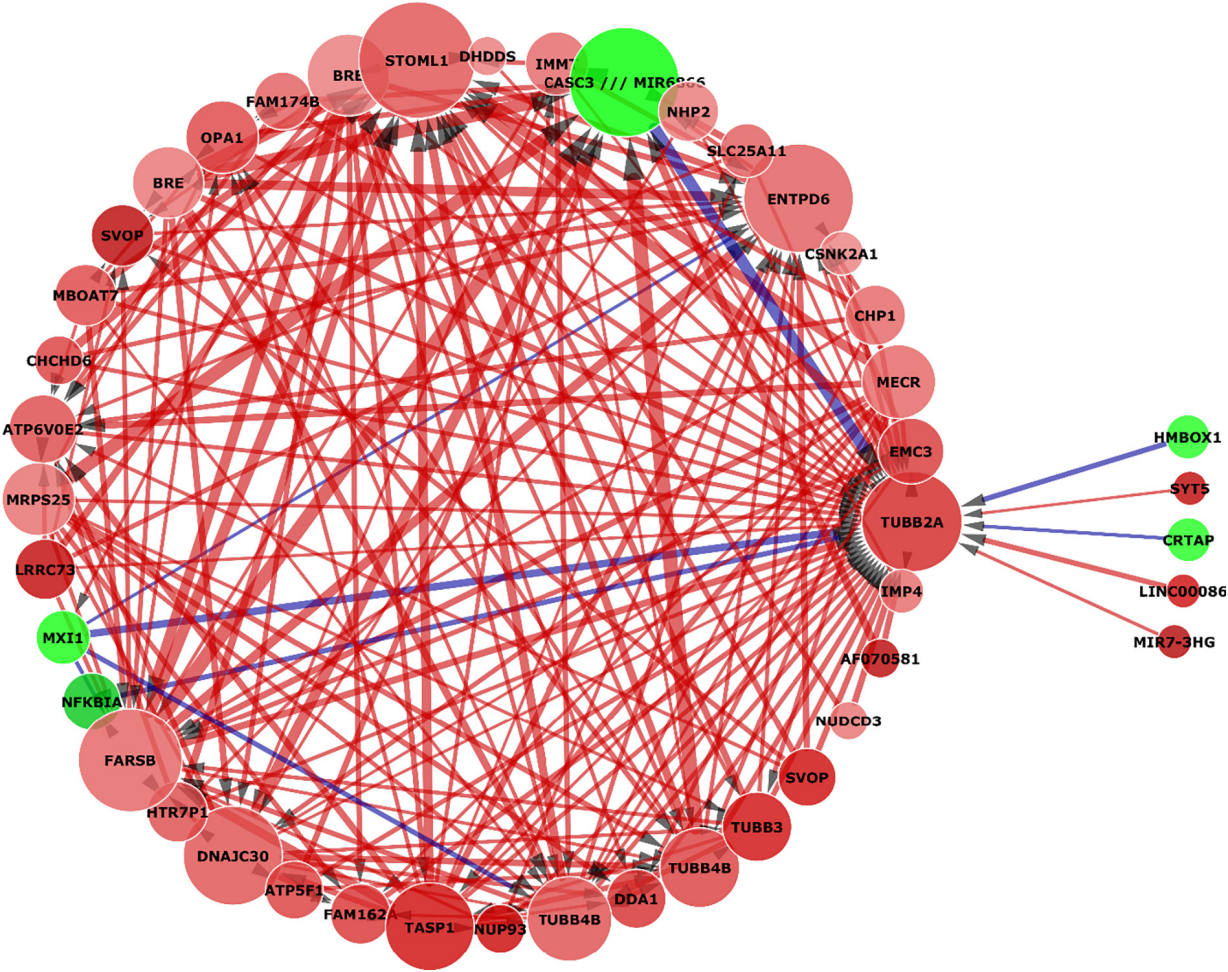
Focused Tubulin interactome based on Fig. 7. Tubulin beta 2A interactions in AD. Of note is its positive regulation by an NFKB inhibitor.

Additionally, one of the major advantages of this method is the that it generates a large and complex interactome that can be used to further examine a gene of interest as seen in Fig. 8.

In this example tubulin 2 beta (TUBB2A), a structural component of microtubules and a gene closely associated with tau, has consistently been in the top genes identified in AD across multiple tests. Due to the size of the previous interactome, there is enough complexity to be able to further analyse the way it interacts with other genes without having to use the algorithm again. If enough genes are identified as relevant to the question, then they can be used as predictors in the continuous ANN and then used for network inference. This also solves the major disadvantage of this methodology; it is computationally expensive and slow.

In Fig. 8 we can observe that TUBB2A is underexpressed but also downregulated by the clear majority of predicted interactions, including by other tubulin variants such as TUBB3 and TUBB4B as well as BRE which was discussed earlier. It is interesting however that both CASC3 and NFKBIA, both of which are overexpressed in this case, are attempting to upregulate TUBB2A, weakly in the case of NFKBIA but relatively strongly in the case of CASC3. CASC3 also appears to be very strongly downregulated by TUBB4B, MRPS25 a mitochondrial ribosomal subunit involved in mitochondrial translation and organelle maintenance and biosynthesis, and FARSB, a Phenylalanyl-TRNA Synthetase Beta Subunit involved in tRNA aminoacylation and has been found to be associated with muscular dystrophy. Thus, it is possible to surmise that the dysregulated state of the TUBB2A gene in the network is directly correlated with mechanistic dysregulations in other genes that in turn affect genes responsible for regulation of TUBB2A itself. CASC3 and NFKBIA are failing to significantly upregulate TUBB2A back to normal levels due to dysregulation within themselves.

### 2.6 Driver Analysis

One of the challenges faced when trying to elucidate a marker, driver or therapy target is the selection criteria used. It is crucial to point out that the data used in these experiments presents us with a “snapshot” of the condition investigated, a generalized picture of how each gene is affected by every other gene, while the biological system is in a state of imbalance. As a result, the biggest hubs of most interactomes tend to be either the genes most up- or down-regulated in the network at the time. This has two potential interpretations. The hub is the source of the imbalance and thus, the most likely driver of the disease and target for therapy, and the downregulation is a result of the system attempting to restore balance, or that the hub is the factor preventing the imbalance by working against the disease and is being upregulated in an effort to restore the system to its original state.

The purpose of the driver analysis is to provide a non-biased selection condition based on the sum of the weights each gene exerts on the network, quantifying the amount of influence on a target and the amount of influence of a target. As explained in Section 2.4 the interaction algorithm analyses the selected genes in a pairwise manner and assigns each of those pairs a value predicting how strongly their genes interact. Hence, by summing the weight that each source gene exerts on each target and vice versa, it becomes possible to rank them by which ones have the greatest overall effect on the network and which ones are the most affected.

The advantage of this method is the fact that it considers and gives equal importance to non-hubs as it only measures the total effect each gene has on the totality of the network. As such, it is possible to draw attention to genes with a multitude of weak interactions rather than only a few strong ones, which might otherwise not be visible. It is reasonable to assume that such genes may not be the greatest drivers of the disease, but crucial components of the system, and this method allows us to analyse those genes without them being obscured by the hubs and most likely drivers, thus giving a wider and impartial view of the condition. Moreover, the driver analysis is not affected by the complexity of the question, being able to provide comparable results across multiple datasets, in both focused and general conditions.

The driver analysis was carried out on the 500 selected genes of the matrix interaction. The most influential source genes showed significant similarities and differences to the results of previous analyses on AD (Table 1). Genes identified in the interactome such as a ITPKB and CASC3 as well as trafficking proteins like TRAK1 and kinases like PRKD3 are expected. Of note is the disproportionate presence of BCL2 when compared to the interactome. However, the sources of interest include RHOBTB3, a member of the highly conserve family of Rho GTPases similar to RHOQ discovered during earlier testing, as well as SRGAP1. SRGAP1 encodes for a GTPase activator and works in conjunction to CDC42, a GTPase of the same family, to negatively regulate neuronal cell migration. Moreover, when combined with receptor ROBO1, it can deactivate CDC42. Its presence so high on the source list as a downregulating factor, indicates that its function is being stronger than expected, resulting in slower cell migration and impediment of the regeneration process. CARD8, discussed earlier, has a strong, negative effect on the network, suppressing the expression of related genes.

**Table 1:**
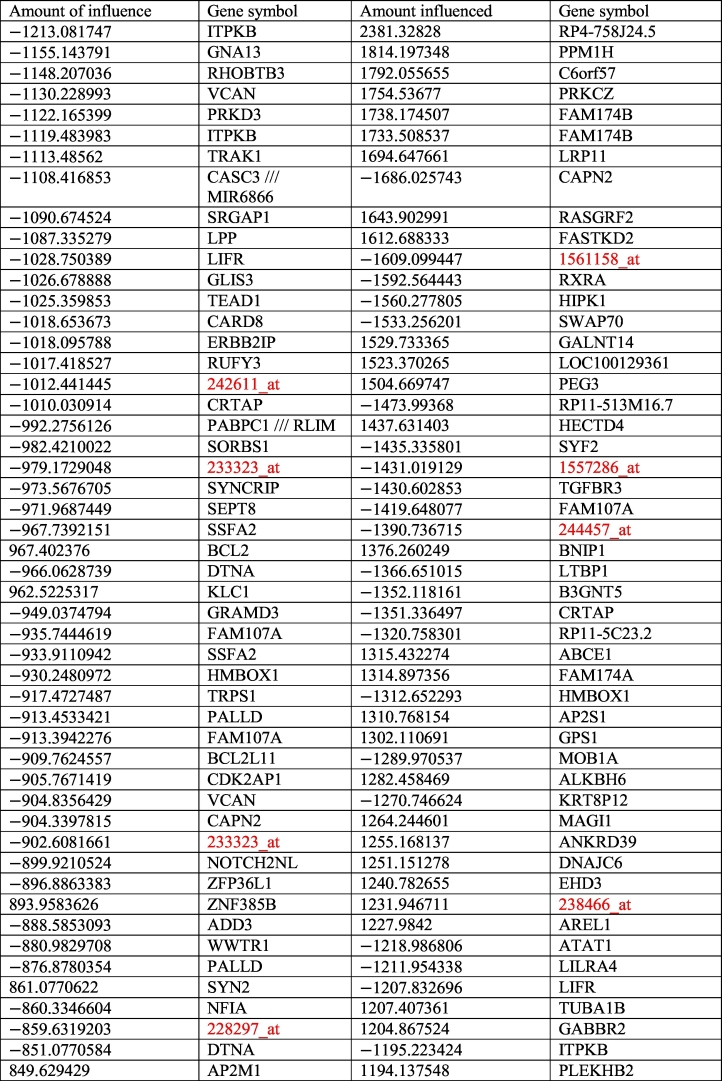
Diver analysis showing the top 50 most influential and most influenced genes according to their unbiased impact on the network in the hippocampus in AD. The influence amount is the sum of all weights calculated by the interaction algorithm and is relative to the rest of the values. Probe IDs in red have not been annotated as of January 2017.

Meanwhile, the most targeted genes on the network include PPM1H, a protein phosphatase, TGFBR3, multiple kinases, and an alpha-tubulin TUBA1B. More beta tubulins are included in the complete list. Also, although rarely seen, ATAT1, an alpha tubulin acetyltransferase, a neuronal cell component crucial to the microtubule growth appears to be negatively regulate. ATAT1 is involved in coenzyme binding and tubulin *N*-acetyltransferase activity and only acetylates older microtubules, being unable to act on unstable ones. Genes such as APGAT1 which fulfil similar purposes have been discovered in previous test, suggesting that slower/weaker acetylation of older microtubules could play a key role in the development of AD. Curiously, one of the upregulated factors is AREL1, apoptosis resistant e3 ubiquitin protein ligase 1, which inhibits apoptosis. It is possible that it is being upregulated in an attempt to keep the neurons alive and functioning to prevent further damage. Finally, the presence of ITPKB as both a significant source and target indicate that it is a crucial component of the system regardless of disease state. The results will be used for a functional analysis via the Bioconductor R package [45]. A second table regarding the driver analysis of the cohort of cognitively normal controls is available in the Supplemental Table 2 for comparison.

## 3 Conclusions and Future Developments

In conclusion, the results obtained by this series of experiments show promise for a greater understanding of the biology behind Alzheimer's disease, its progression and the mechanisms involved. By expanding to other brain regions and datasets and focusing the questions on the most relevant genes, it is possible to identify new markers and drivers of the disease that can be used alongside the current ones to improve prognosis and provide more targets for therapy.

It is worth noting that the results obtained and analysed with this pipeline have been generated without using a null hypothesis, in a non-parametric manner. The only question was the difference between AD and healthy brains and was expanded to include predictors as general as the presence of the disease down to the expression of individual genes. It is evident by the results that by reducing the bias introduced by datamining for very focused questions and increasing the variance, we are presented with multiple potential biomarkers as well as new discovery routes such as further evidence of the role on inflammation and microtubule stabilisation. The pipeline has thus managed to generate unbiased, varied and novel information that can be used to guide further, more targeted research as well as validation of these results experimentally.

Future development will focus on improving the speed and power of the algorithms and increase the interpretability of the results. Using general-purpose computing on graphics processing units, it is possible to reduce the time requirements by up to 75% at the cost of computational power, though recent advances in the field have made it significantly more likely and affordable. Further tests are being focused on the variance between different brain regions as well as the effect of individual genes on the system. Moreover, this series of tests is being repeated in RNA-seq and proteomic datasets in order to study the effect of AD pre and post translation, as well as other gene expression datasets to ensure consistency in the results.

The following are the supplementary data related to this article.

Alzheimer's Hippocampus Driver Analysis. Supplemental Table 1

Cognitively Normal Hippocampus Driver Analysis. Supplemental Table 2

Stepwise and Interaction Algorithm Flowcharts. Supplemental Fig. 1
