# Supplementary material for: An Artificial Neural Network Integrated Pipeline for Biomarker Discovery Using Alzheimer's Disease as a Case Study
Source: Comput Struct Biotechnol J. 2018 Feb 21;16:77–87. doi: 10.1016/j.csbj.2018.02.001 (PMC6026215; doi:10.1016/j.csbj.2018.02.001)
Supplement: Supplementary file 1 — Alzheimer's Hippocampus Driver Analysis. [file mmc1.docx]

| Most Influential | Sum | Gene Symbol | Most Influenced | Sum | Gene Symbol |
| --- | --- | --- | --- | --- | --- |
| 203723_at | -1213.08 | ITPKB | **228835_at** | 2381.328 | RP4-758J24.5 |
| 224761_at | -1155.14 | GNA13 | **212686_at** | 1814.197 | PPM1H |
| 240111_at | -1148.21 | RHOBTB3 | **238504_at** | 1792.056 | C6orf57 |
| 221731_x_at | -1130.23 | VCAN | **202178_at** | 1754.537 | PRKCZ |
| 222565_s_at | -1122.17 | PRKD3 | **221880_s_at** | 1738.175 | FAM174B |
| 235213_at | -1119.48 | ITPKB | **51158_at** | 1733.509 | FAM174B |
| 214924_s_at | -1113.49 | TRAK1 | **225060_at** | 1694.648 | LRP11 |
| 207842_s_at | -1108.42 | CASC3 /// MIR6866 | **208683_at** | -1686.03 | CAPN2 |
| 233888_s_at | -1090.67 | SRGAP1 | **228109_at** | 1643.903 | RASGRF2 |
| 224811_at | -1087.34 | LPP | **229135_at** | 1612.688 | FASTKD2 |
| 225575_at | -1028.75 | LIFR | **1561158_at** | -1609.1 | --- |
| 230258_at | -1026.68 | GLIS3 | **202449_s_at** | -1592.56 | RXRA |
| 224955_at | -1025.36 | TEAD1 | **212291_at** | -1560.28 | HIPK1 |
| 1554479_a_at | -1018.65 | CARD8 | **210369_at** | -1533.26 | SWAP70 |
| 222473_s_at | -1018.1 | ERBB2IP | **219271_at** | 1529.733 | GALNT14 |
| 213437_at | -1017.42 | RUFY3 | **221847_at** | 1523.37 | LOC100129361 |
| 242611_at | -1012.44 | --- | **209242_at** | 1504.67 | PEG3 |
| 1555889_a_at | -1010.03 | CRTAP | **244066_at** | -1473.99 | RP11-513M16.7 |
| 215823_x_at | -992.276 | PABPC1 /// RLIM | **211034_s_at** | 1437.631 | HECTD4 |
| 218087_s_at | -982.421 | SORBS1 | **202553_s_at** | -1435.34 | SYF2 |
| 233323_at | -979.173 | --- | **1557286_at** | -1431.02 | --- |
| 217832_at | -973.568 | SYNCRIP | **204731_at** | -1430.6 | TGFBR3 |
| 208999_at | -971.969 | SEPT8 | **207547_s_at** | -1419.65 | FAM107A |
| 236207_at | -967.739 | SSFA2 | **244457_at** | -1390.74 | --- |
| 203685_at | -967.402 | BCL2 | **37226_at** | 1376.26 | BNIP1 |
| 227084_at | -966.063 | DTNA | **202728_s_at** | -1366.65 | LTBP1 |
| 212878_s_at | 962.5225 | KLC1 | **1554835_a_at** | -1352.12 | B3GNT5 |
| 238049_at | -949.037 | GRAMD3 | **1555889_a_at** | -1351.34 | CRTAP |
| 209074_s_at | -935.744 | FAM107A | **236334_at** | -1320.76 | RP11-5C23.2 |
| 202506_at | -933.911 | SSFA2 | **201873_s_at** | 1315.432 | ABCE1 |
| 225504_at | -930.248 | HMBOX1 | **226752_at** | 1314.897 | FAM174A |
| 222651_s_at | -917.473 | TRPS1 | **225504_at** | -1312.65 | HMBOX1 |
| 200897_s_at | -913.453 | PALLD | **202120_x_at** | 1310.768 | AP2S1 |
| 207547_s_at | -913.394 | FAM107A | **217782_s_at** | 1302.111 | GPS1 |
| 1553096_s_at | -909.762 | BCL2L11 | **214812_s_at** | -1289.97 | MOB1A |
| 201938_at | -905.767 | CDK2AP1 | **225969_at** | 1282.458 | ALKBH6 |
| 204620_s_at | -904.836 | VCAN | **222060_at** | -1270.75 | KRT8P12 |
| 208683_at | -904.34 | CAPN2 | **1559257_a_at** | 1264.245 | MAGI1 |
| 233036_at | -902.608 | --- | **223532_at** | 1255.168 | ANKRD39 |
| 214722_at | -899.921 | NOTCH2NL | **204720_s_at** | 1251.151 | DNAJC6 |
| 211962_s_at | -896.886 | ZFP36L1 | **218935_at** | 1240.783 | EHD3 |
| 1555800_at | 893.9584 | ZNF385B | **238466_at** | 1231.947 | --- |
| 201753_s_at | -888.585 | ADD3 | **202128_at** | 1227.984 | AREL1 |
| 202133_at | -880.983 | WWTR1 | **228510_at** | -1218.99 | ATAT1 |
| 200907_s_at | -876.878 | PALLD | **210313_at** | -1211.95 | LILRA4 |
| 1553037_a_at | 861.0771 | SYN2 | **225575_at** | -1207.83 | LIFR |
| 224970_at | -860.335 | NFIA | **211072_x_at** | 1207.407 | TUBA1B |
| 228297_at | -859.632 | --- | **211679_x_at** | 1204.868 | GABBR2 |
| 211493_x_at | -851.077 | DTNA | **203723_at** | -1195.22 | ITPKB |
| 200613_at | 849.6294 | AP2M1 | **201410_at** | 1194.138 | PLEKHB2 |
| 210742_at | -844.645 | CDC14A | **213558_at** | 1189.313 | PCLO |
| 233877_at | -841.269 | --- | **207026_s_at** | 1187.019 | ATP2B3 |
| 202947_s_at | -833.906 | GYPC | **200906_s_at** | -1163 | PALLD |
| 228510_at | -829.201 | ATAT1 | **224895_at** | -1158.8 | YAP1 |
| 1557286_at | -822.125 | --- | **225719_s_at** | 1157.466 | MRPL55 |
| 222099_s_at | -818.766 | LSM14A | **200078_s_at** | 1150.807 | ATP6V0B |
| 202449_s_at | -814.14 | RXRA | **213793_s_at** | 1145.536 | HOMER1 |
| 208430_s_at | -811.874 | DTNA | **237173_at** | -1135.25 | LOC100132057 |
| 209297_at | -796.759 | ITSN1 | **205646_s_at** | -1126.02 | PAX6 |
| 1555961_a_at | 789.0598 | HINT1 | **208850_s_at** | 1116.566 | THY1 |
| 214762_at | 788.4035 | ATP6V1G2 | **218248_at** | -1103.81 | FAM111A |
| 1554592_a_at | 783.5094 | SLC1A6 | **216056_at** | -1101.82 | CD44 |
| 205609_at | -783.167 | ANGPT1 | **1553211_at** | -1094.91 | ANKFN1 |
| 202822_at | -779.246 | LPP | **238049_at** | -1093 | GRAMD3 |
| 236440_at | 773.7309 | NETO1 | **235213_at** | -1091.26 | ITPKB |
| 210369_at | -773.41 | SWAP70 | **242611_at** | -1080.06 | --- |
| 207501_s_at | 772.3142 | FGF12 | **1553096_s_at** | -1070.02 | BCL2L11 |
| 212877_at | 769.6674 | KLC1 | **219907_at** | 1065.975 | FRS3 |
| 222699_s_at | -763.244 | PLEKHF2 | **218477_at** | -1052.44 | TMEM14A |
| 214436_at | 758.6259 | FBXL2 | **230547_at** | 1050.732 | KCNC1 |
| 1569054_at | -747.552 | SLC1A3 | **203567_s_at** | -1049.23 | TRIM38 |
| 215728_s_at | 746.3263 | ACOT7 | **210108_at** | 1028.265 | CACNA1D |
| 202553_s_at | -744.399 | SYF2 | **211887_x_at** | -1025.06 | MSR1 |
| 218706_s_at | -743.191 | GRAMD3 | **223213_s_at** | -1024.74 | ZHX1 |
| 202016_at | 739.1671 | MEST | **201938_at** | -1022.31 | CDK2AP1 |
| 209569_x_at | 738.8964 | NSG1 | **202133_at** | -1009.11 | WWTR1 |
| 236638_at | 734.7293 | AMER3 | **210650_s_at** | 999.072 | PCLO |
| 35776_at | -732.039 | ITSN1 | **202506_at** | -997.801 | SSFA2 |
| 208752_x_at | -725.482 | NAP1L1 | **211986_at** | -995.64 | AHNAK |
| 210736_x_at | -724.24 | DTNA | **209297_at** | -993.083 | ITSN1 |
| 230773_at | 723.7377 | ZNF385D | **210040_at** | 989.1969 | SLC12A5 |
| 1555801_s_at | 717.4269 | ZNF385B | **205348_s_at** | 984.3187 | DYNC1I1 |
| 210315_at | 716.6529 | SYN2 | **201753_s_at** | -973.297 | ADD3 |
| 200802_at | 715.967 | SARS | **201387_s_at** | 968.0705 | UCHL1 |
| 228063_s_at | 715.5084 | NAP1L5 | **218706_s_at** | -961.33 | GRAMD3 |
| 210617_at | -714.851 | PHEX | **222473_s_at** | -960.595 | ERBB2IP |
| 200906_s_at | -714.767 | PALLD | **212683_at** | 957.4879 | SLC25A44 |
| 232791_at | -713.217 | --- | **202822_at** | -947.195 | LPP |
| 208457_at | 709.0376 | GABRD | **232688_at** | -947.184 | BMP2K |
| 1564706_s_at | 708.7342 | GLS2 | **204514_at** | -937.02 | DPH2 |
| 202728_s_at | -701.813 | LTBP1 | **204521_at** | 928.0489 | FAM216A |
| 236334_at | -700.491 | RP11-5C23.2 | **235066_at** | 923.0736 | MAP4 |
| 244111_at | 691.8854 | KRT222 | **204117_at** | -910.502 | PREP |
| 229039_at | 691.7892 | SYN2 | **218087_s_at** | -907.11 | SORBS1 |
| 230498_at | 671.5373 | MCHR1 | **232377_at** | 906.9702 | NXPH1 |
| 229770_at | 669.9027 | GLT1D1 | **240942_at** | -905.001 | MPHOSPH8 |
| 228062_at | 669.5382 | NAP1L5 | **210742_at** | -903.773 | CDC14A |
| 218248_at | -669.332 | FAM111A | **229300_at** | 902.1661 | RAB3C |
| 1555313_a_at | 668.6704 | MCF2 | **201757_at** | 898.1112 | NDUFS5 /// RPL10 |
| 235656_s_at | 668.0472 | --- | **236207_at** | -895.025 | SSFA2 |
| 218824_at | 667.8046 | PNMAL1 | **222125_s_at** | 893.9448 | P4HTM |
| 240532_at | 667.1965 | SLC32A1 | **1554479_a_at** | -891.501 | CARD8 |
| 202233_s_at | 664.9802 | UQCRH /// UQCRHL | **238569_at** | -883.772 | GABBR1 |
| 201972_at | 664.4244 | ATP6V1A | **222834_s_at** | -878.447 | GNG12 |
| 208308_s_at | 658.5528 | GPI | **204620_s_at** | -868.369 | VCAN |
| 220794_at | 658.4838 | GREM2 | **209991_x_at** | 867.7213 | GABBR2 |
| 207772_s_at | 657.7788 | PRMT8 | **242583_at** | 863.1492 | STON2 |
| 201174_s_at | 654.3957 | TERF2IP | **207153_s_at** | -847.858 | GLMN |
| 236465_at | 654.311 | RNF175 | **213149_at** | -843.978 | DLAT |
| 229925_at | 651.6048 | SLC6A17 | **222099_s_at** | -831.762 | LSM14A |
| 212291_at | -649.482 | HIPK1 | **231986_at** | 825.9801 | RIMS1 |
| 209029_at | 649.3249 | COPS7A | **223708_at** | 824.351 | C1QTNF4 |
| 211685_s_at | 645.5515 | NCALD | **202132_at** | -820.648 | WWTR1 |
| 227662_at | -645.197 | SYNPO2 | **202779_s_at** | 817.3581 | UBE2S |
| 204731_at | -644.439 | TGFBR3 | **207842_s_at** | -816.092 | CASC3 /// MIR6866 |
| 227669_at | 640.7275 | MPC2 | **219896_at** | 816.0201 | CALY |
| 204933_s_at | -640.238 | TNFRSF11B | **208752_x_at** | -807.129 | NAP1L1 |
| 201313_at | 640.1619 | ENO2 | **200960_x_at** | 803.8709 | CLTA |
| 1561158_at | -639.377 | --- | **228680_at** | 795.7601 | KIF3A |
| 202961_s_at | 639.1496 | ARMC2-AS1 /// ATP5J2 | **226086_at** | 792.5487 | SYT13 |
| 207508_at | 637.7393 | ATP5G3 | **203999_at** | 790.3905 | SYT1 |
| 232688_at | -636.456 | BMP2K | **241399_at** | 787.5748 | FAM19A2 |
| 208826_x_at | 635.6861 | HINT1 | **214170_x_at** | 783.5296 | FH |
| 208845_at | 634.387 | VDAC3 | **224761_at** | -776.991 | GNA13 |
| 213553_x_at | -633.977 | APOC1 | **204721_s_at** | 774.6801 | DNAJC6 |
| 222834_s_at | -633.886 | GNG12 | **239765_at** | 773.847 | CPEB3 |
| 205608_s_at | -633.101 | ANGPT1 | **35776_at** | -770.214 | ITSN1 |
| 207721_x_at | 632.9605 | HINT1 | **203724_s_at** | 761.9616 | RUFY3 |
| 225111_s_at | 631.4911 | NAPB | **232791_at** | -747.879 | --- |
| 202941_at | 630.3908 | NDUFV2 | **203685_at** | -746.564 | BCL2 |
| 210313_at | -626.437 | LILRA4 | **203094_at** | 743.2222 | MAD2L1BP |
| 238569_at | -620.104 | GABBR1 | **217077_s_at** | 739.2467 | GABBR2 |
| 208751_at | 616.47 | NAPA | **200897_s_at** | -728.712 | PALLD |
| 209545_s_at | -616.017 | RIPK2 | **213938_at** | 728.3936 | ERC2 |
| 226826_at | 613.9951 | LSM11 | **208999_at** | -727.802 | Sep-08 |
| 213293_s_at | -613.244 | TRIM22 | **209074_s_at** | -722.395 | FAM107A |
| 202395_at | 611.988 | LOC101930324 /// NSF | **232003_at** | 722.1106 | PNMAL2 |
| 230151_at | 608.7842 | SPRYD7 | **1556940_at** | 715.3439 | LOC283484 |
| 203610_s_at | -607.298 | TRIM38 | **211780_x_at** | 711.7324 | DCTN1 |
| 200982_s_at | 606.5216 | ANXA6 | **213386_at** | 702.4629 | TMEM246 |
| 200786_at | 606.5043 | PSMB7 | **204141_at** | 698.7414 | TUBB2A |
| 211825_s_at | -603.774 | FLI1 | **222651_s_at** | -695.841 | TRPS1 |
| 1556159_at | 601.9641 | --- | **223518_at** | -694.958 | DFFA |
| 221908_at | 601.5992 | RNFT2 | **229770_at** | 691.4696 | GLT1D1 |
| 226188_at | 598.5075 | LGALSL | **210736_x_at** | -688.473 | DTNA |
| 213333_at | 595.9023 | MDH2 | **203157_s_at** | 687.8052 | GLS |
| 204365_s_at | 594.4984 | REEP1 | **203000_at** | 684.0876 | STMN2 |
| 211071_s_at | 594.3335 | MLLT11 | **243998_at** | 682.0451 | KRT222 |
| 238462_at | 587.7885 | UBASH3B | **222513_s_at** | -681.776 | SORBS1 |
| 224869_s_at | 587.3067 | MRPS25 | **227401_at** | -680.245 | IL17D |
| 208074_s_at | 586.6726 | AP2S1 | **214078_at** | 678.9538 | AF070581 |
| 202132_at | -586.357 | WWTR1 | **213921_at** | 678.3264 | SST |
| 206984_s_at | 584.3967 | RIT2 | **213437_at** | -676.059 | RUFY3 |
| 203567_s_at | -582.898 | TRIM38 | **244688_at** | 673.9641 | --- |
| 223213_s_at | -582.483 | ZHX1 | **238673_at** | 671.8608 | SAMD12 |
| 235066_at | 580.263 | MAP4 | **219532_at** | 669.3447 | ELOVL4 |
| 212041_at | 580.0242 | ATP6V0D1 | **203517_at** | 666.2342 | MTX2 |
| 208678_at | 579.6262 | ATP6V1E1 | **209902_at** | 665.4726 | ATR |
| 203339_at | 579.5986 | SLC25A12 | **202395_at** | 665.0639 | LOC101930324 /// NSF |
| 200093_s_at | 579.1173 | HINT1 | **232148_at** | -664.862 | NSMAF |
| 212508_at | 578.7059 | MOAP1 | **244463_at** | 660.9286 | ADAM23 |
| 201387_s_at | 578.5783 | UCHL1 | **219752_at** | 658.8935 | RASAL1 |
| 224895_at | -578.473 | YAP1 | **217286_s_at** | 658.265 | NDRG3 |
| 211986_at | -575.185 | AHNAK | **224888_at** | 656.5233 | EPT1 |
| 244457_at | -574.219 | --- | **214998_at** | 652.9472 | AAK1 |
| 242470_at | 570.0049 | EID2B | **204933_s_at** | -649.17 | TNFRSF11B |
| 235540_at | -569.96 | GNRH1 | **203610_s_at** | -648.134 | TRIM38 |
| 212242_at | 567.9714 | TUBA4A | **222699_s_at** | -642.503 | PLEKHF2 |
| 213921_at | 565.8928 | SST | **211047_x_at** | 641.3021 | AP2S1 |
| 200978_at | 565.3378 | MDH1 | **224378_x_at** | 632.9414 | MAP1LC3A |
| 219203_at | 561.7508 | EMC9 | **235656_s_at** | 631.4345 | --- |
| 211047_x_at | 561.1055 | AP2S1 | **1554755_a_at** | 630.2849 | MTUS2 |
| 214812_s_at | -560.74 | MOB1A | **204744_s_at** | -629.077 | IARS |
| 215052_at | 560.4711 | FRMPD4 | **204465_s_at** | 625.6696 | INA |
| 203340_s_at | 558.3332 | SLC25A12 | **205795_at** | 624.8757 | NRXN3 |
| 223529_at | 557.7887 | SYT4 | **207593_at** | 619.8513 | ABCG4 |
| 214260_at | 557.3378 | COPS8 | **224811_at** | -618.729 | LPP |
| 208002_s_at | 553.8907 | ACOT7 | **202927_at** | 616.8147 | PIN1 |
| 227226_at | 552.6221 | MRAP2 | **236277_at** | 613.0187 | AF070581 |
| 210149_s_at | 551.5478 | ATP5H | **223550_s_at** | 609.4759 | CA10 |
| 218300_at | -549.929 | PAGR1 | **227455_at** | 609.1942 | C6orf136 |
| 213451_x_at | -548.212 | TNXA /// TNXB | **219894_at** | 607.2952 | MAGEL2 |
| 204964_s_at | -547.146 | SSPN | **209935_at** | 605.1474 | ATP2C1 |
| 237173_at | -547.005 | LOC100132057 | **233888_s_at** | -601.948 | SRGAP1 |
| 242876_at | -546.258 | AKT3 | **209796_s_at** | 599.9521 | CNPY2 |
| 205646_s_at | -542.152 | PAX6 | **219660_s_at** | 596.7831 | ATP8A2 |
| 208869_s_at | 541.87 | GABARAPL1 | **223604_at** | -592.996 | GARNL3 |
| 205751_at | 541.2557 | SH3GL2 | **223011_s_at** | -591.22 | OCIAD1 |
| 221909_at | 540.6045 | RNFT2 | **219203_at** | 591.1096 | EMC9 |
| 201322_at | 540.2769 | ATP5B | **230773_at** | 589.206 | ZNF385D |
| 208813_at | 539.6017 | GOT1 | **219659_at** | 588.3733 | ATP8A2 |
| 238719_at | 538.9404 | PPP2CA | **228262_at** | 587.0067 | MAP7D2 |
| 213558_at | 537.9431 | PCLO | **224955_at** | -586.067 | TEAD1 |
| 202077_at | 536.3325 | NDUFAB1 | **205531_s_at** | 583.341 | GLS2 |
| 222513_s_at | -535.763 | SORBS1 | **224458_at** | 581.7807 | TMEM246 |
| 202854_at | 535.5354 | HPRT1 | **244739_at** | 579.0529 | RDX |
| 212383_at | 534.3476 | ATP6V0A1 | **203001_s_at** | 577.171 | STMN2 |
| 218163_at | 534.2745 | MCTS1 | **201962_s_at** | 575.5479 | RNF41 |
| 215440_s_at | 531.0412 | BEX4 | **238462_at** | 569.4623 | UBASH3B |
| 217286_s_at | 530.1348 | NDRG3 | **216903_s_at** | 560.1598 | MICU1 |
| 206046_at | 526.8101 | ADAM23 | **229039_at** | 556.3237 | SYN2 |
| 210232_at | 521.7732 | CDC42 | **203607_at** | 556.2603 | INPP5F |
| 232426_at | 520.4085 | SV2B | **226649_at** | 555.5153 | PANK1 |
| 208898_at | 520.3369 | ATP6V1D | **215052_at** | 555.2845 | FRMPD4 |
| 226647_at | 519.1731 | TMEM25 | **236440_at** | 548.659 | NETO1 |
| 204953_at | 518.5381 | SNAP91 | **1557820_at** | 545.9279 | AFG3L2 |
| 210040_at | 516.1374 | SLC12A5 | **213553_x_at** | -545.436 | APOC1 |
| 233337_s_at | 515.4779 | SEZ6L2 | **223367_at** | 544.8331 | DNAJC30 |
| 207507_s_at | 514.4219 | ATP5G3 | **1556151_at** | -544.468 | ITFG1 |
| 212425_at | 514.1983 | SCAMP1 | **221805_at** | 543.4569 | NEFL |
| 230458_at | 512.8617 | SLC45A1 | **206369_s_at** | -534.098 | PIK3CG |
| 1554524_a_at | 512.3119 | OLFM3 | **226470_at** | 533.676 | GGT7 |
| 213268_at | 512.31 | CAMTA1 | **201519_at** | -530.712 | TOMM70A |
| 229300_at | 511.8847 | RAB3C | **225535_s_at** | 529.824 | TIMM23 /// TIMM23B |
| 231763_at | 510.5481 | POLR3A | **233323_at** | -527.026 | --- |
| 225779_at | 510.4205 | SLC27A4 | **223529_at** | 523.8441 | SYT4 |
| 1568603_at | 509.9809 | CADPS | **208969_at** | 523.6801 | NDUFA9 |
| 243317_at | 509.8543 | AX746627 | **201082_s_at** | 522.4415 | DCTN1 /// SLC4A5 |
| 201293_x_at | 509.3059 | LOC101060363 /// PPIA | **230258_at** | -521.58 | GLIS3 |
| 49077_at | 507.71 | PPME1 | **214157_at** | -520.654 | GNAS |
| 1554593_s_at | 506.8041 | SLC1A6 | **210617_at** | -518.008 | PHEX |
| 216333_x_at | -505.796 | TNXA /// TNXB | **225817_at** | -516.02 | CGNL1 /// LOC101930344 /// LOC101930349 |
| 232003_at | 505.298 | PNMAL2 | **223239_at** | 515.1614 | GSKIP |
| 201757_at | 504.2841 | NDUFS5 /// RPL10 | **244647_at** | 512.9227 | WBP11 |
| 213938_at | 503.9022 | ERC2 | **225111_s_at** | 509.2567 | NAPB |
| 225485_at | 503.8816 | CEP41 | **202016_at** | 503.0683 | MEST |
| 223174_at | 502.7295 | BTBD10 | **225781_at** | 496.9104 | MAPK9 |
| 226568_at | 501.7294 | FAM102B | **205278_at** | 495.473 | GAD1 |
| 227702_at | 501.4681 | CYP4X1 | **213451_x_at** | -493.735 | TNXA /// TNXB |
| 216323_x_at | 501.2349 | TUBA3C /// TUBA3D | **221731_x_at** | -489.408 | VCAN |
| 211765_x_at | 496.8382 | PPIA | **230137_at** | -488.779 | TMEM155 |
| 232011_s_at | 496.5484 | MAP1LC3A | **201241_at** | -487.544 | DDX1 |
| 223041_at | 496.2135 | CD99L2 | **229267_at** | 484.1969 | ANAPC1 /// LOC730268 |
| 222005_s_at | 494.888 | GNG3 | **203069_at** | 482.4247 | SV2A |
| 210786_s_at | -492.048 | FLI1 | **241758_at** | 481.9756 | NUP93 |
| 202927_at | 490.6765 | PIN1 | **229649_at** | 480.5208 | NRXN3 |
| 219659_at | 485.8975 | ATP8A2 | **202698_x_at** | -479.653 | COX4I1 |
| 228579_at | 485.2712 | KCNQ3 | **238115_at** | 479.2961 | DNAJC18 |
| 223367_at | 481.0806 | DNAJC30 | **215021_s_at** | 476.62 | NRXN3 |
| 203157_s_at | 479.2539 | GLS | **219301_s_at** | 474.5526 | CNTNAP2 |
| 205278_at | 477.9531 | GAD1 | **215440_s_at** | 474.036 | BEX4 |
| 204749_at | 477.0203 | NAP1L3 | **213333_at** | 472.725 | MDH2 |
| 208451_s_at | -473.07 | C4A /// C4B /// C4B_2 | **214924_s_at** | -469.32 | TRAK1 |
| 201962_s_at | 471.6822 | RNF41 | **202961_s_at** | 463.7806 | ARMC2-AS1 /// ATP5J2 |
| 204465_s_at | 465.6787 | INA | **243317_at** | 458.4486 | AX746627 |
| 231935_at | 465.2298 | ARPP21 | **218300_at** | -456.892 | PAGR1 |
| 206343_s_at | 465.1019 | NRG1 | **201431_s_at** | -456.197 | DPYSL3 |
| 208870_x_at | 464.1798 | ATP5C1 | **226813_at** | -451.46 | NTPCR |
| 238115_at | 462.0177 | DNAJC18 | **1556034_s_at** | -450.75 | MTMR11 |
| 217841_s_at | 460.3279 | PPME1 | **214434_at** | 450.4019 | HSPA12A |
| 207636_at | -459.959 | SERPINI2 | **232011_s_at** | 447.6499 | MAP1LC3A |
| 210532_s_at | 458.9324 | C14orf2 | **208451_s_at** | -439.407 | C4A /// C4B /// C4B_2 |
| 201082_s_at | 458.4674 | DCTN1 /// SLC4A5 | **204001_at** | 439.1362 | SNAPC3 |
| 213366_x_at | 456.7975 | ATP5C1 | **230458_at** | 432.4125 | SLC45A1 |
| 219688_at | 456.4745 | BBS7 | **207984_s_at** | 424.7349 | MPP2 |
| 212661_x_at | 451.8994 | LOC101060363 /// PPIA | **217942_at** | 421.8627 | MRPS35 |
| 209036_s_at | 451.7223 | MDH2 | **232426_at** | 421.6899 | SV2B |
| 209570_s_at | 448.4802 | NSG1 | **227702_at** | 421.3509 | CYP4X1 |
| 208969_at | 446.6259 | NDUFA9 | **203020_at** | 420.4294 | RABGAP1L |
| 219896_at | 446.5716 | CALY | **230708_at** | 420.1529 | PRICKLE1 |
| 214434_at | 445.6782 | HSPA12A | **202041_s_at** | -413.998 | FIBP |
| 232195_at | 444.0725 | GPR158 | **203889_at** | 413.7113 | SCG5 |
| 37950_at | 443.9679 | PREP | **226188_at** | 407.0311 | LGALSL |
| 1554755_a_at | 442.5282 | MTUS2 | **222565_s_at** | -406.495 | PRKD3 |
| 208846_s_at | 439.6988 | VDAC3 | **227226_at** | 406.1829 | MRAP2 |
| 207853_s_at | 439.672 | SNCB | **215728_s_at** | 405.2953 | ACOT7 |
| 214998_at | 439.1024 | AAK1 | **209029_at** | 403.9069 | COPS7A |
| 211072_x_at | 438.3777 | TUBA1B | **226653_at** | 403.3984 | MARK1 |
| 213486_at | 437.2015 | COPG2IT1 | **212661_x_at** | 403.2344 | LOC101060363 /// PPIA |
| 227455_at | 436.4778 | C6orf136 | **203396_at** | 402.2869 | PSMA4 |
| 213278_at | 435.9007 | MTMR9 | **205257_s_at** | 400.3495 | AMPH |
| 205257_s_at | 434.0623 | AMPH | **227461_at** | -397.883 | STON2 |
| 209991_x_at | 433.1299 | GABBR2 | **210532_s_at** | 395.3816 | C14orf2 |
| 217976_s_at | 432.6682 | DYNC1LI1 | **222005_s_at** | 394.4984 | GNG3 |
| 227461_at | -429.93 | STON2 | **204749_at** | 392.4232 | NAP1L3 |
| 229526_at | 428.6714 | AQP11 | **213270_at** | 390.2275 | MPP2 |
| 233910_at | 425.3976 | TMEFF2 | **217832_at** | -389.639 | SYNCRIP |
| 227401_at | -424.994 | IL17D | **205608_s_at** | -385.995 | ANGPT1 |
| 209540_at | 424.7923 | IGF1 | **231869_at** | 385.2574 | KIAA1586 |
| 209186_at | 421.6858 | ATP2A2 | **229526_at** | 383.6497 | AQP11 |
| 206404_at | 421.1105 | FGF9 | **218568_at** | -382.975 | AGK |
| 204141_at | 420.5649 | TUBB2A | **224970_at** | -381.596 | NFIA |
| 213808_at | 419.2197 | ADAM23 | **227176_at** | 376.8269 | SLC2A13 |
| 222060_at | -417.313 | KRT8P12 | **213278_at** | 375.6296 | MTMR9 |
| 225817_at | -415.969 | CGNL1 /// LOC101930344 /// LOC101930349 | **206408_at** | -375.53 | LRRTM2 |
| 244688_at | 413.3407 | --- | **204953_at** | 374.1472 | SNAP91 |
| 635_s_at | 411.7843 | PPP2R5B | **229925_at** | 372.7854 | SLC6A17 |
| 219894_at | 410.4889 | MAGEL2 | **209545_s_at** | -370.083 | RIPK2 |
| 205348_s_at | 410.2197 | DYNC1I1 | **226259_at** | 367.2636 | EXOC6 |
| 205531_s_at | 410.0155 | GLS2 | **202683_s_at** | 366.6368 | RNMT |
| 214157_at | 408.7995 | GNAS | **226822_at** | -362.708 | STOX2 |
| 207026_s_at | 408.1714 | ATP2B3 | **215143_at** | -360.452 | DPY19L2P2 |
| 227219_x_at | 407.9944 | MAP1LC3A | **242876_at** | -359.362 | AKT3 |
| 223461_at | 406.0109 | TBC1D7 | **223093_at** | 358.829 | ANKH |
| 230137_at | 405.995 | TMEM155 | **218597_s_at** | -358.62 | CISD1 |
| 210247_at | 405.2493 | SYN2 | **219983_at** | -357.454 | HRASLS |
| 203001_s_at | 404.8228 | STMN2 | **210149_s_at** | 357.0469 | ATP5H |
| 232377_at | 403.2976 | NXPH1 | **235852_at** | -352.803 | STON2 |
| 218720_x_at | 401.1462 | SEZ6L2 | **224913_s_at** | 352.4561 | TIMM50 |
| 203607_at | 400.1375 | INPP5F | **233877_at** | -351.503 | --- |
| 203517_at | 399.7382 | MTX2 | **228297_at** | 349.0066 | --- |
| 220334_at | 398.1892 | RGS17 | **210247_at** | 348.0113 | SYN2 |
| 203999_at | 397.6325 | SYT1 | **239935_at** | 345.658 | MDGA2 |
| 203094_at | 393.3066 | MAD2L1BP | **232195_at** | 345.0181 | GPR158 |
| 215021_s_at | 392.3398 | NRXN3 | **212987_at** | 344.777 | FBXO9 |
| 216903_s_at | 391.0412 | MICU1 | **227456_s_at** | -340.583 | C6orf136 |
| 203000_at | 390.3201 | STMN2 | **201293_x_at** | 338.3984 | LOC101060363 /// PPIA |
| 213386_at | 388.5349 | TMEM246 | **225485_at** | 338.2935 | CEP41 |
| 224378_x_at | 388.3577 | MAP1LC3A | **211765_x_at** | 331.0401 | PPIA |
| 219532_at | 386.3311 | ELOVL4 | **208846_s_at** | 331.0231 | VDAC3 |
| 202120_x_at | 385.7374 | AP2S1 | **202712_s_at** | 328.8745 | CKMT1A /// CKMT1B |
| 244066_at | -385.371 | RP11-513M16.7 | **213720_s_at** | 328.3299 | SMARCA4 |
| 224888_at | 384.7065 | EPT1 | **208870_x_at** | 327.4047 | ATP5C1 |
| 220889_s_at | 384.6269 | CA10 | **1568603_at** | 326.9598 | CADPS |
| 219660_s_at | 382.0809 | ATP8A2 | **203862_s_at** | 323.9078 | ACTN2 |
| 1553211_at | -379.449 | ANKFN1 | **225841_at** | -323.88 | HENMT1 |
| 1556034_s_at | -378.573 | MTMR11 | **209003_at** | 321.7179 | SLC25A11 |
| 244463_at | 378.174 | ADAM23 | **244099_at** | 320.6363 | CACNG2 |
| 223708_at | 378.0153 | C1QTNF4 | **202078_at** | 319.0476 | COPS3 |
| 236277_at | 375.5994 | AF070581 | **218760_at** | 317.5592 | COQ6 |
| 226086_at | 374.8411 | SYT13 | **209444_at** | -316.023 | RAP1GDS1 |
| 202779_s_at | 374.5728 | UBE2S | **213366_x_at** | 314.8865 | ATP5C1 |
| 242583_at | -374.058 | STON2 | **208898_at** | 309.8831 | ATP6V1D |
| 204001_at | 370.8504 | SNAPC3 | **220334_at** | 309.8622 | RGS17 |
| 210108_at | 370.7908 | CACNA1D | **205012_s_at** | 307.9528 | HAGH |
| 201714_at | 368.7893 | TUBG1 | **201714_at** | 302.6311 | TUBG1 |
| 227176_at | 365.3517 | SLC2A13 | **211962_s_at** | -299.101 | ZFP36L1 |
| 214230_at | 364.9168 | CDC42 | **226154_at** | 298.4369 | DNM1L |
| 218477_at | 364.1236 | TMEM14A | **216323_x_at** | 293.5372 | TUBA3C /// TUBA3D |
| 225781_at | 360.7321 | MAPK9 | **208017_s_at** | -293.446 | MCF2 |
| 218597_s_at | 360.2695 | CISD1 | **213293_s_at** | -291.913 | TRIM22 |
| 216056_at | -359.448 | CD44 | **231120_x_at** | 291.8384 | PKIB |
| 213884_s_at | 358.0184 | TRIM3 | **213217_at** | -291.32 | ADCY2 |
| 202078_at | 357.1325 | COPS3 | **210232_at** | 288.6277 | CDC42 |
| 218760_at | 356.7027 | COQ6 | **202233_s_at** | 288.6219 | UQCRH /// UQCRHL |
| 217979_at | 356.4424 | TSPAN13 | **207853_s_at** | 287.2982 | SNCB |
| 223340_at | 355.5172 | ATL1 | **236838_at** | 283.2905 | SRCIN1 |
| 205230_at | 354.4 | RPH3A | **227084_at** | -282.427 | DTNA |
| 228262_at | 350.3152 | MAP7D2 | **211685_s_at** | 281.7687 | NCALD |
| 205012_s_at | 346.2004 | HAGH | **213884_s_at** | 281.3861 | TRIM3 |
| 223550_s_at | 343.5668 | CA10 | **205609_at** | 279.251 | ANGPT1 |
| 209694_at | 339.2039 | PTS | **226568_at** | 271.7044 | FAM102B |
| 223532_at | 338.1822 | ANKRD39 | **221908_at** | 268.3859 | RNFT2 |
| 213217_at | -337.965 | ADCY2 | **205230_at** | 265.0923 | RPH3A |
| 221880_s_at | 332.3428 | FAM174B | **244111_at** | 264.7226 | KRT222 |
| 232148_at | -331.605 | NSMAF | **1552736_a_at** | 264.1744 | NETO1 |
| 202683_s_at | 331.5663 | RNMT | **206404_at** | 260.6328 | FGF9 |
| 235664_at | -331.14 | --- | **212041_at** | 257.78 | ATP6V0D1 |
| 209935_at | -330.499 | ATP2C1 | **207636_at** | 257.4378 | SERPINI2 |
| 235852_at | -330.282 | STON2 | **200982_s_at** | 256.8772 | ANXA6 |
| 205359_at | 329.0578 | AKAP6 | **202947_s_at** | -254.722 | GYPC |
| 214665_s_at | 326.6452 | CHP1 | **235006_at** | 254.2419 | CDKN2AIPNL |
| 202712_s_at | 324.0712 | CKMT1A /// CKMT1B | **205751_at** | 250.7806 | SH3GL2 |
| 238889_at | 322.3045 | AGBL5 | **207507_s_at** | 250.1977 | ATP5G3 |
| 217077_s_at | 319.7116 | GABBR2 | **210786_s_at** | -248.987 | FLI1 |
| 203020_at | 317.2382 | RABGAP1L | **1556159_at** | 248.1631 | --- |
| 229097_at | -315.779 | DIAPH3 | **228579_at** | 247.8002 | KCNQ3 |
| 200960_x_at | 313.6386 | CLTA | **204365_s_at** | 247.0526 | REEP1 |
| 206369_s_at | -312.661 | PIK3CG | **220794_at** | 243.6546 | GREM2 |
| 229649_at | 310.7752 | NRXN3 | **217979_at** | 243.4581 | TSPAN13 |
| 218901_at | -310.21 | PLSCR4 | **214722_at** | -241.201 | NOTCH2NL |
| 241399_at | 309.7083 | FAM19A2 | **241801_at** | -237.019 | PGAP1 |
| 229267_at | 307.7823 | ANAPC1 /// LOC730268 | **231763_at** | 236.9518 | POLR3A |
| 1556940_at | 306.567 | LOC283484 | **240111_at** | -235.188 | RHOBTB3 |
| 1554835_a_at | -305.413 | B3GNT5 | **235253_at** | 230.8349 | RAD1 |
| 223093_at | 304.3867 | ANKH | **200978_at** | 229.2484 | MDH1 |
| 223011_s_at | 303.4819 | OCIAD1 | **208869_s_at** | 227.461 | GABARAPL1 |
| 201519_at | 301.5185 | TOMM70A | **208430_s_at** | 226.1252 | DTNA |
| 207593_at | 301.1088 | ABCG4 | **242470_at** | 225.8199 | EID2B |
| 203069_at | 301.0595 | SV2A | **223530_at** | -221.24 | TDRKH |
| 203862_s_at | 299.2924 | ACTN2 | **1554592_a_at** | 220.5799 | SLC1A6 |
| 210650_s_at | 298.6419 | PCLO | **224869_s_at** | 218.2891 | MRPS25 |
| 213720_s_at | 290.3598 | SMARCA4 | **203339_at** | -217.837 | SLC25A12 |
| 202041_s_at | 288.6445 | FIBP | **209694_at** | 215.6529 | PTS |
| 227444_at | 288.2494 | ARMCX4 | **220889_s_at** | 215.5907 | CA10 |
| 228680_at | 283.4397 | KIF3A | **227219_x_at** | -212.677 | MAP1LC3A |
| 224913_s_at | 282.9273 | TIMM50 | **226339_at** | 209.2648 | TRUB1 |
| 221805_at | 281.1573 | NEFL | **239265_at** | 208.653 | SLC35G1 |
| 213270_at | 280.8989 | MPP2 | **218720_x_at** | 207.499 | SEZ6L2 |
| 228109_at | 279.6226 | RASGRF2 | **37950_at** | 206.8818 | PREP |
| 204521_at | 279.3037 | FAM216A | **216333_x_at** | -206.512 | TNXA /// TNXB |
| 226653_at | 278.7971 | MARK1 | **201174_s_at** | 205.0268 | TERF2IP |
| 226259_at | 278.1819 | EXOC6 | **202854_at** | 204.4983 | HPRT1 |
| 221847_at | 276.1608 | LOC100129361 | **227662_at** | 199.8726 | SYNPO2 |
| 209003_at | 275.9778 | SLC25A11 | **214230_at** | 193.2752 | CDC42 |
| 238466_at | 274.5517 | --- | **223461_at** | 192.6564 | TBC1D7 |
| 211615_s_at | 273.3865 | LRPPRC | **213268_at** | 191.4669 | CAMTA1 |
| 201241_at | 272.5821 | DDX1 | **212072_s_at** | -190.634 | CSNK2A1 |
| 244099_at | 267.8911 | CACNG2 | **218969_at** | 190.0734 | PAM16 |
| 201431_s_at | -267.395 | DPYSL3 | **1555313_a_at** | -190.008 | MCF2 |
| 202825_at | 265.1569 | SLC25A4 | **228062_at** | 186.2206 | NAP1L5 |
| 238504_at | 264.774 | C6orf57 | **235664_at** | -180.701 | --- |
| 244739_at | 263.4616 | RDX | **209186_at** | 180.1734 | ATP2A2 |
| 214170_x_at | 262.693 | FH | **227669_at** | 180.0577 | MPC2 |
| 226470_at | 256.7807 | GGT7 | **226647_at** | 178.8719 | TMEM25 |
| 223239_at | 255.264 | GSKIP | **236638_at** | 173.6254 | AMER3 |
| 203724_s_at | 254.1272 | RUFY3 | **214306_at** | -172.892 | OPA1 |
| 211887_x_at | -251.552 | MSR1 | **209540_at** | 172.6241 | IGF1 |
| 226154_at | 248.6084 | DNM1L | **242317_at** | -166.801 | HIGD1A |
| 214306_at | 240.047 | OPA1 | **214665_s_at** | 164.719 | CHP1 |
| 225060_at | 238.7873 | LRP11 | **209990_s_at** | 163.4001 | GABBR2 |
| 222125_s_at | 238.6281 | P4HTM | **208457_at** | 161.8416 | GABRD |
| 218671_s_at | 236.9508 | ATPIF1 | **1569054_at** | -161.213 | SLC1A3 |
| 204471_at | 235.7707 | GAP43 | **212508_at** | 160.2722 | MOAP1 |
| 203889_at | 234.6158 | SCG5 | **49077_at** | 159.7288 | PPME1 |
| 224458_at | 234.3127 | TMEM246 | **233337_s_at** | 159.2456 | SEZ6L2 |
| 226813_at | 232.9356 | NTPCR | **217841_s_at** | 157.6807 | PPME1 |
| 204744_s_at | 232.5088 | IARS | **202077_at** | 154.1901 | NDUFAB1 |
| 230708_at | 232.0271 | PRICKLE1 | **204964_s_at** | -152.057 | SSPN |
| 243998_at | 229.3549 | KRT222 | **1554593_s_at** | 149.9104 | SLC1A6 |
| 202698_x_at | 227.9036 | COX4I1 | **1555801_s_at** | 149.087 | ZNF385B |
| 209902_at | 227.2711 | ATR | **212877_at** | 148.826 | KLC1 |
| 225969_at | 226.75 | ALKBH6 | **217976_s_at** | 148.0934 | DYNC1LI1 |
| 202486_at | 226.5041 | AFG3L2 | **208308_s_at** | 147.7353 | GPI |
| 219271_at | -225.499 | GALNT14 | **208074_s_at** | 147.6278 | AP2S1 |
| 51158_at | 222.9417 | FAM174B | **214762_at** | 147.3905 | ATP6V1G2 |
| 204117_at | 221.7192 | PREP | **201972_at** | 147.0541 | ATP6V1A |
| 215143_at | 217.1009 | DPY19L2P2 | **218901_at** | -144.644 | PLSCR4 |
| 200078_s_at | 213.4137 | ATP6V0B | **207508_at** | 140.0341 | ATP5G3 |
| 213793_s_at | 212.4751 | HOMER1 | **206343_s_at** | -139.257 | NRG1 |
| 205795_at | 206.9964 | NRXN3 | **229097_at** | -138.881 | DIAPH3 |
| 235253_at | 202.0353 | RAD1 | **238719_at** | 136.5449 | PPP2CA |
| 235006_at | 199.6572 | CDKN2AIPNL | **228063_s_at** | 135.8384 | NAP1L5 |
| 214078_at | 198.3174 | AF070581 | **208813_at** | 135.3045 | GOT1 |
| 226752_at | 194.8728 | FAM174A | **204471_at** | 134.9463 | GAP43 |
| 212987_at | 191.0975 | FBXO9 | **212242_at** | 134.7068 | TUBA4A |
| 203745_at | 190.5071 | HCCS | **208826_x_at** | 132.3672 | HINT1 |
| 213427_at | 185.6025 | RPP40 | **212383_at** | 131.4903 | ATP6V0A1 |
| 219752_at | 183.5263 | RASAL1 | **206046_at** | 130.5282 | ADAM23 |
| 231120_x_at | 182.435 | PKIB | **205359_at** | 129.5228 | AKAP6 |
| 238673_at | 179.5717 | SAMD12 | **214260_at** | -125.745 | COPS8 |
| 219301_s_at | 177.2748 | CNTNAP2 | **1555961_a_at** | 125.7431 | HINT1 |
| 225719_s_at | 176.1604 | MRPL55 | **209589_s_at** | 125.7246 | EPHB2 |
| 212686_at | -175.668 | PPM1H | **240532_at** | 125.1146 | SLC32A1 |
| 206408_at | 174.4097 | LRRTM2 | **233910_at** | -122.714 | TMEFF2 |
| 203396_at | 173.6381 | PSMA4 | **200907_s_at** | -121.286 | PALLD |
| 239935_at | 171.1344 | MDGA2 | **209569_x_at** | -119.326 | NSG1 |
| 230547_at | 170.7616 | KCNC1 | **213486_at** | 116.1213 | COPG2IT1 |
| 209589_s_at | -170.591 | EPHB2 | **208678_at** | 113.8205 | ATP6V1E1 |
| 219208_at | 169.1046 | FBXO11 | **218824_at** | -112.358 | PNMAL1 |
| 211034_s_at | 165.9004 | HECTD4 | **208845_at** | -111.993 | VDAC3 |
| 212072_s_at | 164.2272 | CSNK2A1 | **201322_at** | 111.8173 | ATP5B |
| 202178_at | 164.1665 | PRKCZ | **223174_at** | 111.5716 | BTBD10 |
| 211780_x_at | 154.1744 | DCTN1 | **211615_s_at** | 111.2908 | LRPPRC |
| 204721_s_at | -154.143 | DNAJC6 | **236465_at** | 111.1069 | RNF175 |
| 239765_at | 154.0876 | CPEB3 | **227468_at** | 110.566 | CPT1C |
| 223518_at | 153.6948 | DFFA | **206984_s_at** | 109.0803 | RIT2 |
| 1556151_at | 151.9731 | ITFG1 | **218671_s_at** | 108.7828 | ATPIF1 |
| 226767_s_at | 149.8617 | FAHD1 | **223340_at** | 108.2118 | ATL1 |
| 231967_at | 147.9648 | PHF20L1 | **200786_at** | 106.8577 | PSMB7 |
| 241801_at | 146.9874 | PGAP1 | **238871_at** | 104.7532 | MLLT4 |
| 238658_at | 143.9744 | --- | **1554524_a_at** | 104.4727 | OLFM3 |
| 208850_s_at | 143.633 | THY1 | **225779_at** | 104.3554 | SLC27A4 |
| 209796_s_at | 141.6818 | CNPY2 | **208751_at** | 103.6941 | NAPA |
| 238871_at | 139.7666 | MLLT4 | **238889_at** | -102.327 | AGBL5 |
| 201873_s_at | -137.021 | ABCE1 | **211825_s_at** | -100.912 | FLI1 |
| 209242_at | 134.339 | PEG3 | **200093_s_at** | 99.18889 | HINT1 |
| 207153_s_at | 132.8518 | GLMN | **200613_at** | 98.79305 | AP2M1 |
| 223530_at | 127.9521 | TDRKH | **230151_at** | 95.1775 | SPRYD7 |
| 217782_s_at | 127.3692 | GPS1 | **207501_s_at** | -85.1758 | FGF12 |
| 211679_x_at | 126.5163 | GABBR2 | **218163_at** | 84.58546 | MCTS1 |
| 240942_at | 125.0154 | MPHOSPH8 | **214436_at** | 84.53391 | FBXL2 |
| 201410_at | -124.947 | PLEKHB2 | **1554133_at** | 84.28382 | RUFY2 |
| 209444_at | 121.9157 | RAP1GDS1 | **208002_s_at** | 82.90369 | ACOT7 |
| 225535_s_at | 112.2326 | TIMM23 /// TIMM23B | **213808_at** | 80.6313 | ADAM23 |
| 213149_at | 111.8264 | DLAT | **231935_at** | 74.56355 | ARPP21 |
| 227468_at | 109.4208 | CPT1C | **221909_at** | 70.21675 | RNFT2 |
| 207984_s_at | 101.092 | MPP2 | **1553037_a_at** | 69.70476 | SYN2 |
| 212683_at | 94.91348 | SLC25A44 | **200802_at** | 69.66659 | SARS |
| 244647_at | 89.85304 | WBP11 | **203340_s_at** | 68.47243 | SLC25A12 |
| 202128_at | -85.3391 | AREL1 | **212425_at** | -66.8776 | SCAMP1 |
| 231869_at | 84.51993 | KIAA1586 | **230498_at** | 59.0723 | MCHR1 |
| 227456_s_at | 81.79146 | C6orf136 | **213427_at** | 56.80502 | RPP40 |
| 242317_at | 79.67675 | HIGD1A | **635_s_at** | 49.87908 | PPP2R5B |
| 223604_at | 76.76849 | GARNL3 | **233036_at** | -49.7785 | --- |
| 208017_s_at | 75.38065 | MCF2 | **215823_x_at** | -47.625 | PABPC1 /// RLIM |
| 218935_at | 71.15767 | EHD3 | **201313_at** | -45.4282 | ENO2 |
| 219907_at | 61.38719 | FRS3 | **202486_at** | 44.76528 | AFG3L2 |
| 229135_at | 60.88453 | FASTKD2 | **226767_s_at** | 43.19214 | FAHD1 |
| 225841_at | 60.02535 | HENMT1 | **209036_s_at** | 37.67159 | MDH2 |
| 1559257_a_at | 53.16606 | MAGI1 | **211071_s_at** | -36.7858 | MLLT11 |
| 37226_at | -52.6381 | BNIP1 | **207772_s_at** | 36.0643 | PRMT8 |
| 1557820_at | -52.3503 | AFG3L2 | **211493_x_at** | 33.29334 | DTNA |
| 1552736_a_at | 50.68467 | NETO1 | **238658_at** | 31.95698 | --- |
| 239265_at | 49.7943 | SLC35G1 | **235540_at** | -30.2928 | GNRH1 |
| 204720_s_at | -48.125 | DNAJC6 | **227444_at** | -30.0678 | ARMCX4 |
| 209990_s_at | 43.9096 | GABBR2 | **210315_at** | 19.18734 | SYN2 |
| 204514_at | 39.94365 | DPH2 | **202825_at** | -19.1829 | SLC25A4 |
| 219983_at | -33.2648 | HRASLS | **226826_at** | 17.6533 | LSM11 |
| 226339_at | 31.61987 | TRUB1 | **1555800_at** | 17.05508 | ZNF385B |
| 231986_at | 28.64193 | RIMS1 | **203745_at** | 17.01282 | HCCS |
| 226649_at | 21.89886 | PANK1 | **223041_at** | -16.0713 | CD99L2 |
| 236838_at | -17.4106 | SRCIN1 | **212878_s_at** | 15.17748 | KLC1 |
| 241758_at | 14.91252 | NUP93 | **202941_at** | -11.8918 | NDUFV2 |
| 1554133_at | 12.76393 | RUFY2 | **207721_x_at** | 11.03058 | HINT1 |
| 218568_at | -12.1702 | AGK | **231967_at** | -10.0462 | PHF20L1 |
| 218969_at | 11.44138 | PAM16 | **209570_s_at** | 8.630514 | NSG1 |
| 226822_at | -10.2288 | STOX2 | **1564706_s_at** | -7.54456 | GLS2 |
| 217942_at | 9.993471 | MRPS35 | **219208_at** | 7.440692 | FBXO11 |
| 228835_at | 3.254713 | RP4-758J24.5 | **219688_at** | -1.64909 | BBS7 |
