# Supplementary material for: An Artificial Neural Network Integrated Pipeline for Biomarker Discovery Using Alzheimer's Disease as a Case Study
Source: Comput Struct Biotechnol J. 2018 Feb 21;16:77–87. doi: 10.1016/j.csbj.2018.02.001 (PMC6026215; doi:10.1016/j.csbj.2018.02.001)
Supplement: Supplementary file 2 — Cognitively Normal Hippocampus Driver Analysis. [file mmc2.docx]

| Most Influential | Sum | Gene Symbol | Most Influenced | Sum | Gene Symbol |
| --- | --- | --- | --- | --- | --- |
| 224761_at | -2409.38 | GNA13 | **203001_s_at** | 4519.62 | STMN2 |
| 225575_at | -2405.90 | LIFR | **204141_at** | 4058.04 | TUBB2A |
| 203723_at | -2153.59 | ITPKB | **219301_s_at** | 3919.68 | CNTNAP2 |
| 235213_at | -1961.50 | ITPKB | **223367_at** | 3888.02 | DNAJC30 |
| 218706_s_at | -1938.31 | GRAMD3 | **202712_s_at** | 3732.35 | CKMT1A /// CKMT1B |
| 221731_x_at | -1924.91 | VCAN | **221805_at** | 3697.77 | NEFL |
| 222473_s_at | -1922.68 | ERBB2IP | **203000_at** | 3645.04 | STMN2 |
| 1557286_at | -1911.60 | --- | **225535_s_at** | 3479.56 | TIMM23 /// TIMM23B |
| 1554479_a_at | -1895.43 | CARD8 | **205278_at** | 3350.46 | GAD1 |
| 201753_s_at | -1868.75 | ADD3 | **203999_at** | 3214.15 | SYT1 |
| 200613_at | 1859.31 | AP2M1 | **202961_s_at** | 3213.12 | ARMC2-AS1 /// ATP5J2 |
| 225504_at | -1828.94 | HMBOX1 | **219532_at** | 2988.75 | ELOVL4 |
| 202506_at | -1808.65 | SSFA2 | **215440_s_at** | 2839.72 | BEX4 |
| 215823_x_at | -1778.58 | PABPC1 /// RLIM | **237173_at** | -2594.89 | LOC100132057 |
| 212508_at | 1744.55 | MOAP1 | **228063_s_at** | 2580.32 | NAP1L5 |
| 215728_s_at | 1721.58 | ACOT7 | **213558_at** | 2559.68 | PCLO |
| 230258_at | -1693.09 | GLIS3 | **218935_at** | 2516.49 | EHD3 |
| 200897_s_at | -1685.90 | PALLD | **204521_at** | 2484.92 | FAM216A |
| 204620_s_at | -1675.53 | VCAN | **210149_s_at** | 2466.58 | ATP5H |
| 222651_s_at | -1657.42 | TRPS1 | **222005_s_at** | 2464.25 | GNG3 |
| 208683_at | -1653.12 | CAPN2 | **223708_at** | 2413.05 | C1QTNF4 |
| 218248_at | -1647.98 | FAM111A | **238504_at** | 2390.11 | C6orf57 |
| 214722_at | -1643.61 | NOTCH2NL | **231986_at** | 2379.43 | RIMS1 |
| 208999_at | -1635.52 | SEPT8 | **242317_at** | 2314.09 | HIGD1A |
| 222565_s_at | -1624.04 | PRKD3 | **208846_s_at** | 2302.75 | VDAC3 |
| 227461_at | -1617.25 | STON2 | **200978_at** | 2223.28 | MDH1 |
| 224970_at | -1616.71 | NFIA | **225485_at** | 2136.62 | CEP41 |
| 210369_at | -1613.26 | SWAP70 | **244111_at** | 2122.08 | KRT222 |
| 202120_x_at | 1606.68 | AP2S1 | **205608_s_at** | -2116.07 | ANGPT1 |
| 233877_at | -1606.22 | --- | **226154_at** | 2086.46 | DNM1L |
| 213333_at | 1596.75 | MDH2 | **209242_at** | -2080.68 | PEG3 |
| 236207_at | -1584.31 | SSFA2 | **202077_at** | 2077.13 | NDUFAB1 |
| 211962_s_at | -1581.24 | ZFP36L1 | **242611_at** | -2035.80 | --- |
| 228680_at | 1577.72 | KIF3A | **202728_s_at** | -2021.68 | LTBP1 |
| 238049_at | -1572.17 | GRAMD3 | **242876_at** | -2013.73 | AKT3 |
| 202132_at | -1570.89 | WWTR1 | **232011_s_at** | 2000.28 | MAP1LC3A |
| 215052_at | 1563.17 | FRMPD4 | **227444_at** | 1974.61 | ARMCX4 |
| 202553_s_at | -1550.16 | SYF2 | **201082_s_at** | 1968.20 | DCTN1 /// SLC4A5 |
| 209074_s_at | -1543.89 | FAM107A | **218597_s_at** | 1961.04 | CISD1 |
| 201241_at | 1542.89 | DDX1 | **241758_at** | 1959.48 | NUP93 |
| 211986_at | -1537.67 | AHNAK | **232148_at** | -1945.48 | NSMAF |
| 207842_s_at | -1535.92 | CASC3 /// MIR6866 | **204471_at** | 1928.30 | GAP43 |
| 219688_at | 1529.93 | BBS7 | **210532_s_at** | 1914.70 | C14orf2 |
| 222099_s_at | -1529.27 | LSM14A | **218568_at** | -1905.41 | AGK |
| 203567_s_at | -1519.09 | TRIM38 | **201410_at** | 1898.73 | PLEKHB2 |
| 240532_at | 1510.46 | SLC32A1 | **202233_s_at** | 1886.58 | UQCRH /// UQCRHL |
| 208813_at | 1506.16 | GOT1 | **211071_s_at** | 1880.61 | MLLT11 |
| 204964_s_at | -1500.45 | SSPN | **226086_at** | 1875.81 | SYT13 |
| 208002_s_at | 1493.10 | ACOT7 | **202395_at** | 1874.33 | LOC101930324 /// NSF |
| 223708_at | 1490.02 | C1QTNF4 | **232791_at** | -1852.85 | --- |
| 224811_at | -1489.57 | LPP | **210040_at** | 1849.12 | SLC12A5 |
| 1555889_a_at | -1482.93 | CRTAP | **238466_at** | 1845.09 | --- |
| 212383_at | 1481.02 | ATP6V0A1 | **214924_s_at** | -1827.21 | TRAK1 |
| 202822_at | -1479.30 | LPP | **207508_at** | 1811.43 | ATP5G3 |
| 1554592_a_at | 1473.03 | SLC1A6 | **207507_s_at** | 1772.06 | ATP5G3 |
| 244066_at | -1452.45 | RP11-513M16.7 | **210232_at** | 1770.25 | CDC42 |
| 227455_at | 1452.36 | C6orf136 | **35776_at** | -1762.83 | ITSN1 |
| 1556940_at | 1444.87 | LOC283484 | **214998_at** | 1737.51 | AAK1 |
| 205751_at | 1435.24 | SH3GL2 | **202947_s_at** | -1732.84 | GYPC |
| 224955_at | -1433.64 | TEAD1 | **225111_s_at** | 1732.31 | NAPB |
| 210742_at | -1432.60 | CDC14A | **200982_s_at** | 1720.96 | ANXA6 |
| 201714_at | 1432.51 | TUBG1 | **202120_x_at** | 1719.37 | AP2S1 |
| 244647_at | 1427.37 | WBP11 | **1553096_s_at** | -1709.14 | BCL2L11 |
| 229770_at | 1425.82 | GLT1D1 | **204953_at** | 1689.97 | SNAP91 |
| 222834_s_at | -1424.31 | GNG12 | **209991_x_at** | 1683.69 | GABBR2 |
| 201387_s_at | 1419.42 | UCHL1 | **203020_at** | -1679.72 | RABGAP1L |
| 202133_at | -1417.18 | WWTR1 | **202927_at** | 1678.85 | PIN1 |
| 214260_at | 1411.17 | COPS8 | **200906_s_at** | -1668.35 | PALLD |
| 201962_s_at | 1408.40 | RNF41 | **203069_at** | 1667.33 | SV2A |
| 208451_s_at | -1404.13 | C4A /// C4B /// C4B_2 | **205348_s_at** | 1658.01 | DYNC1I1 |
| 207772_s_at | 1403.76 | PRMT8 | **224761_at** | -1655.73 | GNA13 |
| 233036_at | -1400.30 | --- | **201519_at** | 1641.89 | TOMM70A |
| 202927_at | 1399.03 | PIN1 | **214762_at** | 1639.83 | ATP6V1G2 |
| 210108_at | 1396.17 | CACNA1D | **213451_x_at** | -1636.32 | TNXA /// TNXB |
| 208457_at | 1394.38 | GABRD | **208898_at** | 1621.08 | ATP6V1D |
| 233337_s_at | 1389.22 | SEZ6L2 | **207593_at** | 1613.40 | ABCG4 |
| 200907_s_at | -1387.24 | PALLD | **214722_at** | -1602.86 | NOTCH2NL |
| 215143_at | 1384.45 | DPY19L2P2 | **218477_at** | -1587.64 | TMEM14A |
| 211679_x_at | 1382.08 | GABBR2 | **211615_s_at** | 1576.26 | LRPPRC |
| 212878_s_at | 1379.18 | KLC1 | **202486_at** | 1572.92 | AFG3L2 |
| 203745_at | 1377.26 | HCCS | **223239_at** | 1565.23 | GSKIP |
| 207547_s_at | -1374.19 | FAM107A | **202132_at** | -1551.40 | WWTR1 |
| 219208_at | 1367.85 | FBXO11 | **207853_s_at** | 1532.88 | SNCB |
| 1564706_s_at | 1366.05 | GLS2 | **212508_at** | 1521.30 | MOAP1 |
| 208308_s_at | 1363.62 | GPI | **203517_at** | 1515.35 | MTX2 |
| 202947_s_at | -1363.04 | GYPC | **203723_at** | -1513.18 | ITPKB |
| 244463_at | 1362.38 | ADAM23 | **233036_at** | -1503.43 | --- |
| 206046_at | 1354.65 | ADAM23 | **224955_at** | -1502.01 | TEAD1 |
| 1556159_at | 1354.08 | --- | **241399_at** | 1498.16 | FAM19A2 |
| 205531_s_at | 1343.15 | GLS2 | **213386_at** | 1490.46 | TMEM246 |
| 223174_at | 1340.77 | BTBD10 | **201387_s_at** | 1488.01 | UCHL1 |
| 217077_s_at | 1334.28 | GABBR2 | **226767_s_at** | 1480.03 | FAHD1 |
| 235253_at | 1332.12 | RAD1 | **218163_at** | 1477.12 | MCTS1 |
| 225781_at | 1326.54 | MAPK9 | **214230_at** | 1472.39 | CDC42 |
| 205257_s_at | 1325.90 | AMPH | **212291_at** | -1465.79 | HIPK1 |
| 236465_at | 1312.43 | RNF175 | **213366_x_at** | 1463.78 | ATP5C1 |
| 201938_at | -1310.79 | CDK2AP1 | **202941_at** | 1458.36 | NDUFV2 |
| 218720_x_at | 1305.01 | SEZ6L2 | **226470_at** | 1452.20 | GGT7 |
| 214812_s_at | -1303.36 | MOB1A | **216056_at** | -1449.74 | CD44 |
| 225817_at | -1303.17 | CGNL1 /// LOC101930344 /// LOC101930349 | **226653_at** | 1447.06 | MARK1 |
| 1555800_at | 1301.58 | ZNF385B | **211887_x_at** | -1427.58 | MSR1 |
| 235066_at | 1299.22 | MAP4 | **228297_at** | -1425.41 | --- |
| 209991_x_at | 1298.78 | GABBR2 | **202016_at** | 1420.61 | MEST |
| 211047_x_at | 1291.28 | AP2S1 | **222699_s_at** | -1408.45 | PLEKHF2 |
| 227669_at | 1288.39 | MPC2 | **233888_s_at** | -1400.38 | SRGAP1 |
| 235852_at | -1274.77 | STON2 | **206369_s_at** | -1398.95 | PIK3CG |
| 227444_at | 1272.03 | ARMCX4 | **202078_at** | 1398.84 | COPS3 |
| 202683_s_at | 1267.34 | RNMT | **214306_at** | 1376.18 | OPA1 |
| 232688_at | -1261.38 | BMP2K | **224811_at** | -1371.96 | LPP |
| 1553096_s_at | -1260.14 | BCL2L11 | **210742_at** | -1356.85 | CDC14A |
| 212041_at | 1257.50 | ATP6V0D1 | **212383_at** | 1349.15 | ATP6V0A1 |
| 208074_s_at | 1256.01 | AP2S1 | **1553211_at** | -1335.22 | ANKFN1 |
| 1553037_a_at | 1255.65 | SYN2 | **206984_s_at** | 1335.08 | RIT2 |
| 204731_at | -1253.46 | TGFBR3 | **201962_s_at** | 1331.66 | RNF41 |
| 229925_at | 1251.96 | SLC6A17 | **215823_x_at** | -1323.99 | PABPC1 /// RLIM |
| 225111_s_at | 1251.72 | NAPB | **203685_at** | -1318.40 | BCL2 |
| 232011_s_at | 1251.34 | MAP1LC3A | **204749_at** | 1311.55 | NAP1L3 |
| 205646_s_at | -1250.28 | PAX6 | **209036_s_at** | 1295.92 | MDH2 |
| 223532_at | 1248.37 | ANKRD39 | **202128_at** | 1292.22 | AREL1 |
| 227084_at | -1247.25 | DTNA | **209935_at** | -1291.32 | ATP2C1 |
| 211034_s_at | 1245.54 | HECTD4 | **211765_x_at** | 1288.53 | PPIA |
| 219894_at | 1237.79 | MAGEL2 | **212877_at** | 1280.05 | KLC1 |
| 213884_s_at | 1235.59 | TRIM3 | **205257_s_at** | 1277.82 | AMPH |
| 230458_at | 1235.54 | SLC45A1 | **232688_at** | -1275.43 | BMP2K |
| 222060_at | -1231.57 | KRT8P12 | **207547_s_at** | -1274.19 | FAM107A |
| 213293_s_at | -1227.46 | TRIM22 | **225841_at** | 1273.08 | HENMT1 |
| 1555961_a_at | 1223.14 | HINT1 | **226339_at** | 1263.01 | TRUB1 |
| 211887_x_at | -1221.67 | MSR1 | **213921_at** | 1256.54 | SST |
| 238462_at | 1219.89 | UBASH3B | **229135_at** | 1254.09 | FASTKD2 |
| 204117_at | 1219.61 | PREP | **1568603_at** | 1252.03 | CADPS |
| 217286_s_at | 1212.51 | NDRG3 | **1556940_at** | 1246.24 | LOC283484 |
| 208751_at | 1211.68 | NAPA | **212878_s_at** | 1245.41 | KLC1 |
| 207636_at | -1210.22 | SERPINI2 | **213278_at** | 1244.90 | MTMR9 |
| 227401_at | -1209.02 | IL17D | **216323_x_at** | 1243.52 | TUBA3C /// TUBA3D |
| 200093_s_at | 1208.86 | HINT1 | **217979_at** | 1240.44 | TSPAN13 |
| 226568_at | 1208.26 | FAM102B | **214157_at** | 1236.19 | GNAS |
| 220889_s_at | 1205.53 | CA10 | **201293_x_at** | 1221.74 | LOC101060363 /// PPIA |
| 204953_at | 1197.34 | SNAP91 | **203157_s_at** | 1212.14 | GLS |
| 200906_s_at | -1194.69 | PALLD | **244066_at** | -1198.13 | RP11-513M16.7 |
| 1569054_at | -1193.68 | SLC1A3 | **229039_at** | 1194.83 | SYN2 |
| 214434_at | 1189.07 | HSPA12A | **227084_at** | -1189.84 | DTNA |
| 217979_at | 1185.59 | TSPAN13 | **1555889_a_at** | -1189.31 | CRTAP |
| 51158_at | 1185.09 | FAM174B | **227669_at** | 1185.86 | MPC2 |
| 214998_at | 1182.36 | AAK1 | **208850_s_at** | 1183.40 | THY1 |
| 1555801_s_at | 1180.09 | ZNF385B | **1554479_a_at** | -1183.04 | CARD8 |
| 49077_at | 1178.80 | PPME1 | **236465_at** | 1172.27 | RNF175 |
| 1561158_at | -1177.35 | --- | **207721_x_at** | 1155.59 | HINT1 |
| 213793_s_at | 1175.47 | HOMER1 | **235066_at** | 1148.20 | MAP4 |
| 202728_s_at | -1172.78 | LTBP1 | **228680_at** | 1147.24 | KIF3A |
| 217841_s_at | 1168.74 | PPME1 | **227468_at** | 1145.06 | CPT1C |
| 202854_at | 1166.13 | HPRT1 | **227702_at** | 1141.02 | CYP4X1 |
| 230708_at | 1161.87 | PRICKLE1 | **225504_at** | -1127.08 | HMBOX1 |
| 233323_at | -1160.09 | --- | **210313_at** | -1125.88 | LILRA4 |
| 202233_s_at | 1159.86 | UQCRH /// UQCRHL | **222651_s_at** | -1122.79 | TRPS1 |
| 214078_at | 1158.44 | AF070581 | **200907_s_at** | -1122.27 | PALLD |
| 215021_s_at | 1157.95 | NRXN3 | **228062_at** | 1118.85 | NAP1L5 |
| 235540_at | -1157.58 | GNRH1 | **202854_at** | 1118.65 | HPRT1 |
| 211071_s_at | 1157.33 | MLLT11 | **208683_at** | -1110.99 | CAPN2 |
| 232003_at | 1153.92 | PNMAL2 | **211679_x_at** | 1103.80 | GABBR2 |
| 1554593_s_at | 1153.43 | SLC1A6 | **238115_at** | -1093.60 | DNAJC18 |
| 227456_s_at | 1152.54 | C6orf136 | **215143_at** | 1091.75 | DPY19L2P2 |
| 223041_at | 1150.57 | CD99L2 | **204465_s_at** | 1088.60 | INA |
| 218824_at | 1150.25 | PNMAL1 | **1555961_a_at** | 1083.47 | HINT1 |
| 224913_s_at | 1149.02 | TIMM50 | **209003_at** | 1083.43 | SLC25A11 |
| 240111_at | -1146.76 | RHOBTB3 | **230151_at** | 1080.51 | SPRYD7 |
| 213386_at | 1144.85 | TMEM246 | **201938_at** | -1080.33 | CDK2AP1 |
| 201313_at | 1139.87 | ENO2 | **212242_at** | 1059.76 | TUBA4A |
| 241801_at | 1139.76 | PGAP1 | **216333_x_at** | -1045.12 | TNXA /// TNXB |
| 205608_s_at | -1138.33 | ANGPT1 | **204731_at** | -1042.62 | TGFBR3 |
| 213427_at | 1131.66 | RPP40 | **219896_at** | 1024.76 | CALY |
| 225779_at | 1130.13 | SLC27A4 | **209545_s_at** | -1023.44 | RIPK2 |
| 212242_at | 1128.94 | TUBA4A | **222473_s_at** | -1019.84 | ERBB2IP |
| 219660_s_at | 1127.90 | ATP8A2 | **235213_at** | -1016.80 | ITPKB |
| 205609_at | -1125.00 | ANGPT1 | **213938_at** | 1015.85 | ERC2 |
| 222005_s_at | 1120.63 | GNG3 | **202683_s_at** | 1015.55 | RNMT |
| 227176_at | 1119.91 | SLC2A13 | **1564706_s_at** | 1013.30 | GLS2 |
| 202041_s_at | 1113.01 | FIBP | **206404_at** | 1011.28 | FGF9 |
| 207853_s_at | 1111.85 | SNCB | **225781_at** | 1008.80 | MAPK9 |
| 227226_at | 1110.27 | MRAP2 | **236838_at** | -1006.47 | SRCIN1 |
| 219659_at | 1109.92 | ATP8A2 | **205795_at** | -1001.28 | NRXN3 |
| 214924_s_at | -1105.72 | TRAK1 | **212686_at** | -997.74 | PPM1H |
| 232377_at | 1104.68 | NXPH1 | **225575_at** | -994.41 | LIFR |
| 207501_s_at | 1104.62 | FGF12 | **210369_at** | -989.70 | SWAP70 |
| 224895_at | -1103.34 | YAP1 | **202553_s_at** | -981.56 | SYF2 |
| 208869_s_at | 1098.81 | GABARAPL1 | **214665_s_at** | 979.06 | CHP1 |
| 218935_at | 1097.64 | EHD3 | **230258_at** | -978.07 | GLIS3 |
| 204521_at | 1095.86 | FAM216A | **226568_at** | 975.99 | FAM102B |
| 205348_s_at | 1092.39 | DYNC1I1 | **213553_x_at** | -973.49 | APOC1 |
| 228510_at | -1090.69 | ATAT1 | **203567_s_at** | -971.67 | TRIM38 |
| 221908_at | 1088.39 | RNFT2 | **204001_at** | 971.58 | SNAPC3 |
| 227219_x_at | 1086.70 | MAP1LC3A | **210247_at** | 969.57 | SYN2 |
| 227702_at | 1086.29 | CYP4X1 | **223340_at** | 963.10 | ATL1 |
| 224458_at | 1086.03 | TMEM246 | **227461_at** | -962.56 | STON2 |
| 203685_at | -1085.73 | BCL2 | **223529_at** | 960.37 | SYT4 |
| 224869_s_at | 1085.51 | MRPS25 | **217077_s_at** | 959.02 | GABBR2 |
| 226188_at | 1084.25 | LGALSL | **202698_x_at** | 948.11 | COX4I1 |
| 225841_at | 1083.49 | HENMT1 | **228510_at** | -946.68 | ATAT1 |
| 204365_s_at | 1083.32 | REEP1 | **242583_at** | -937.25 | STON2 |
| 244111_at | 1076.88 | KRT222 | **225817_at** | -936.02 | CGNL1 /// LOC101930344 /// LOC101930349 |
| 200982_s_at | 1075.83 | ANXA6 | **221909_at** | 934.68 | RNFT2 |
| 239935_at | 1074.96 | MDGA2 | **204964_s_at** | -928.22 | SSPN |
| 200802_at | 1072.84 | SARS | **243998_at** | 927.95 | KRT222 |
| 239765_at | 1072.38 | CPEB3 | **201174_s_at** | 924.25 | TERF2IP |
| 244457_at | -1070.44 | --- | **208869_s_at** | 923.00 | GABARAPL1 |
| 203340_s_at | 1066.77 | SLC25A12 | **202178_at** | 916.34 | PRKCZ |
| 1554755_a_at | 1062.72 | MTUS2 | **208678_at** | 916.24 | ATP6V1E1 |
| 205230_at | 1060.85 | RPH3A | **213268_at** | 909.42 | CAMTA1 |
| 237173_at | -1059.62 | LOC100132057 | **201972_at** | 906.00 | ATP6V1A |
| 230498_at | 1055.51 | MCHR1 | **238569_at** | -905.12 | GABBR1 |
| 209540_at | 1048.51 | IGF1 | **218248_at** | -903.55 | FAM111A |
| 242611_at | -1045.06 | --- | **239265_at** | 902.95 | SLC35G1 |
| 229039_at | 1044.42 | SYN2 | **209694_at** | 899.26 | PTS |
| 232426_at | 1041.34 | SV2B | **239765_at** | 898.02 | CPEB3 |
| 202486_at | 1040.85 | AFG3L2 | **202822_at** | -895.88 | LPP |
| 213553_x_at | -1035.48 | APOC1 | **218824_at** | 894.53 | PNMAL1 |
| 210232_at | 1034.11 | CDC42 | **203607_at** | 893.81 | INPP5F |
| 203607_at | 1033.95 | INPP5F | **212661_x_at** | 886.15 | LOC101060363 /// PPIA |
| 226086_at | 1030.50 | SYT13 | **203724_s_at** | 882.39 | RUFY3 |
| 210247_at | 1030.09 | SYN2 | **200078_s_at** | 876.93 | ATP6V0B |
| 223340_at | 1029.62 | ATL1 | **201322_at** | 875.12 | ATP5B |
| 236334_at | -1025.77 | RP11-5C23.2 | **209540_at** | 874.31 | IGF1 |
| 212877_at | 1025.63 | KLC1 | **202449_s_at** | -873.33 | RXRA |
| 202178_at | 1024.96 | PRKCZ | **224458_at** | 860.58 | TMEM246 |
| 221909_at | 1020.08 | RNFT2 | **232426_at** | 857.85 | SV2B |
| 209029_at | 1019.21 | COPS7A | **236440_at** | 856.71 | NETO1 |
| 207593_at | 1016.86 | ABCG4 | **238719_at** | 854.45 | PPP2CA |
| 211780_x_at | 1015.66 | DCTN1 | **223604_at** | -853.45 | GARNL3 |
| 210040_at | 1015.36 | SLC12A5 | **223093_at** | 851.59 | ANKH |
| 227468_at | 1013.71 | CPT1C | **215052_at** | 840.25 | FRMPD4 |
| 209990_s_at | 1013.32 | GABBR2 | **211047_x_at** | 839.62 | AP2S1 |
| 214762_at | 1013.17 | ATP6V1G2 | **210315_at** | 838.29 | SYN2 |
| 200078_s_at | 1012.89 | ATP6V0B | **200897_s_at** | -837.56 | PALLD |
| 202395_at | 1011.92 | LOC101930324 /// NSF | **235540_at** | -834.39 | GNRH1 |
| 230151_at | 1011.45 | SPRYD7 | **228109_at** | 832.18 | RASGRF2 |
| 205278_at | 1008.43 | GAD1 | **1569054_at** | -828.71 | SLC1A3 |
| 235006_at | 1007.40 | CDKN2AIPNL | **240532_at** | 827.92 | SLC32A1 |
| 235656_s_at | 1006.19 | --- | **229300_at** | 823.22 | RAB3C |
| 222513_s_at | -1004.73 | SORBS1 | **218671_s_at** | 822.83 | ATPIF1 |
| 228297_at | -1002.69 | --- | **209074_s_at** | -822.02 | FAM107A |
| 209570_s_at | 1002.41 | NSG1 | **1561158_at** | -814.09 | --- |
| 202078_at | 1001.06 | COPS3 | **226826_at** | 812.23 | LSM11 |
| 208850_s_at | 999.86 | THY1 | **213437_at** | -807.38 | RUFY3 |
| 210313_at | -997.97 | LILRA4 | **204744_s_at** | 805.17 | IARS |
| 223529_at | 996.67 | SYT4 | **1554755_a_at** | 799.75 | MTUS2 |
| 219203_at | 995.76 | EMC9 | **209297_at** | -799.12 | ITSN1 |
| 223530_at | 995.47 | TDRKH | **219203_at** | 797.01 | EMC9 |
| 37950_at | 994.13 | PREP | **200093_s_at** | 794.87 | HINT1 |
| 227662_at | -991.71 | SYNPO2 | **1556034_s_at** | -792.62 | MTMR11 |
| 232148_at | -986.43 | NSMAF | **1557820_at** | 788.23 | AFG3L2 |
| 242583_at | -982.89 | STON2 | **233877_at** | -784.82 | --- |
| 1556034_s_at | -980.30 | MTMR11 | **200786_at** | 778.58 | PSMB7 |
| 210736_x_at | -978.85 | DTNA | **222060_at** | -773.80 | KRT8P12 |
| 203000_at | 977.61 | STMN2 | **224895_at** | -773.56 | YAP1 |
| 203001_s_at | 976.76 | STMN2 | **238658_at** | 773.46 | --- |
| 230547_at | 974.80 | KCNC1 | **230708_at** | 770.95 | PRICKLE1 |
| 231986_at | 974.36 | RIMS1 | **201714_at** | 766.11 | TUBG1 |
| 233888_s_at | -974.06 | SRGAP1 | **226188_at** | 754.66 | LGALSL |
| 202449_s_at | -972.16 | RXRA | **217286_s_at** | 754.28 | NDRG3 |
| 202941_at | 969.18 | NDUFV2 | **217782_s_at** | 753.69 | GPS1 |
| 203094_at | 968.74 | MAD2L1BP | **240942_at** | -752.13 | MPHOSPH8 |
| 231935_at | 965.70 | ARPP21 | **1559257_a_at** | -745.17 | MAGI1 |
| 206984_s_at | 965.61 | RIT2 | **203396_at** | 739.96 | PSMA4 |
| 202779_s_at | 965.07 | UBE2S | **1554835_a_at** | -738.20 | B3GNT5 |
| 229267_at | 964.72 | ANAPC1 /// LOC730268 | **244457_at** | -733.28 | --- |
| 201174_s_at | 962.84 | TERF2IP | **219208_at** | 729.86 | FBXO11 |
| 203157_s_at | 958.34 | GLS | **205751_at** | 722.40 | SH3GL2 |
| 228063_s_at | 957.69 | NAP1L5 | **203889_at** | 713.62 | SCG5 |
| 203610_s_at | -956.87 | TRIM38 | **202041_s_at** | 711.06 | FIBP |
| 244688_at | 955.58 | --- | **209990_s_at** | 707.83 | GABBR2 |
| 211825_s_at | -954.91 | FLI1 | **236334_at** | -700.60 | RP11-5C23.2 |
| 210532_s_at | 948.58 | C14orf2 | **208017_s_at** | -699.42 | MCF2 |
| 216323_x_at | 943.04 | TUBA3C /// TUBA3D | **215728_s_at** | 684.46 | ACOT7 |
| 1568603_at | 938.55 | CADPS | **229267_at** | 684.21 | ANAPC1 /// LOC730268 |
| 201519_at | 938.26 | TOMM70A | **228835_at** | -681.84 | RP4-758J24.5 |
| 226826_at | 937.68 | LSM11 | **205359_at** | 681.50 | AKAP6 |
| 1555313_a_at | 935.86 | MCF2 | **231869_at** | -681.40 | KIAA1586 |
| 208430_s_at | -933.88 | DTNA | **1553037_a_at** | 680.09 | SYN2 |
| 224378_x_at | 933.85 | MAP1LC3A | **201313_at** | 678.54 | ENO2 |
| 211685_s_at | 931.76 | NCALD | **210650_s_at** | 678.38 | PCLO |
| 205359_at | 931.66 | AKAP6 | **209444_at** | -678.04 | RAP1GDS1 |
| 209186_at | 926.80 | ATP2A2 | **230547_at** | 670.45 | KCNC1 |
| 216333_x_at | -919.50 | TNXA /// TNXB | **209589_s_at** | -669.22 | EPHB2 |
| 210315_at | 919.08 | SYN2 | **228579_at** | 667.77 | KCNQ3 |
| 209569_x_at | 916.36 | NSG1 | **235006_at** | 666.60 | CDKN2AIPNL |
| 212683_at | 916.32 | SLC25A44 | **213427_at** | 663.57 | RPP40 |
| 223093_at | 914.36 | ANKH | **222565_s_at** | -660.02 | PRKD3 |
| 222699_s_at | -911.94 | PLEKHF2 | **203340_s_at** | 659.88 | SLC25A12 |
| 231763_at | 909.97 | POLR3A | **212041_at** | 655.94 | ATP6V0D1 |
| 220334_at | 909.46 | RGS17 | **203339_at** | -655.66 | SLC25A12 |
| 230773_at | 909.02 | ZNF385D | **208826_x_at** | 650.47 | HINT1 |
| 213268_at | 905.77 | CAMTA1 | **205012_s_at** | 650.24 | HAGH |
| 213808_at | 903.12 | ADAM23 | **211986_at** | -650.01 | AHNAK |
| 217832_at | -901.47 | SYNCRIP | **224888_at** | 645.43 | EPT1 |
| 238889_at | 901.34 | AGBL5 | **208969_at** | 640.46 | NDUFA9 |
| 1554524_a_at | 901.17 | OLFM3 | **211034_s_at** | 635.26 | HECTD4 |
| 207721_x_at | 898.24 | HINT1 | **217832_at** | -631.00 | SYNCRIP |
| 204744_s_at | 897.06 | IARS | **219983_at** | 624.03 | HRASLS |
| 236277_at | 895.58 | AF070581 | **208074_s_at** | 618.12 | AP2S1 |
| 202077_at | 893.60 | NDUFAB1 | **1554593_s_at** | 613.70 | SLC1A6 |
| 200960_x_at | 892.52 | CLTA | **208308_s_at** | 612.67 | GPI |
| 214230_at | 891.64 | CDC42 | **214812_s_at** | -611.71 | MOB1A |
| 233910_at | 890.56 | TMEFF2 | **230458_at** | 610.69 | SLC45A1 |
| 217976_s_at | 889.89 | DYNC1LI1 | **200802_at** | 606.15 | SARS |
| 202128_at | 887.28 | AREL1 | **208813_at** | 602.46 | GOT1 |
| 228579_at | 880.00 | KCNQ3 | **221908_at** | 602.21 | RNFT2 |
| 219532_at | 879.14 | ELOVL4 | **223041_at** | 602.06 | CD99L2 |
| 204465_s_at | 878.46 | INA | **212683_at** | -595.87 | SLC25A44 |
| 218087_s_at | -876.33 | SORBS1 | **229770_at** | 591.17 | GLT1D1 |
| 211765_x_at | 875.75 | PPIA | **211780_x_at** | 589.92 | DCTN1 |
| 232195_at | 874.44 | GPR158 | **208870_x_at** | 587.82 | ATP5C1 |
| 204471_at | 872.29 | GAP43 | **221731_x_at** | -586.15 | VCAN |
| 214436_at | 867.84 | FBXL2 | **205609_at** | -585.21 | ANGPT1 |
| 210149_s_at | 865.03 | ATP5H | **227456_s_at** | 583.97 | C6orf136 |
| 213437_at | -855.49 | RUFY3 | **209029_at** | 582.53 | COPS7A |
| 235664_at | -854.95 | --- | **208002_s_at** | 580.69 | ACOT7 |
| 207026_s_at | 850.21 | ATP2B3 | **232003_at** | 580.48 | PNMAL2 |
| 209036_s_at | 844.90 | MDH2 | **238049_at** | -574.65 | GRAMD3 |
| 213217_at | -841.51 | ADCY2 | **226647_at** | 574.57 | TMEM25 |
| 211493_x_at | -841.28 | DTNA | **238871_at** | 569.86 | MLLT4 |
| 212987_at | 840.41 | FBXO9 | **209569_x_at** | 565.48 | NSG1 |
| 213486_at | 839.52 | COPG2IT1 | **201873_s_at** | 561.68 | ABCE1 |
| 213451_x_at | -838.29 | TNXA /// TNXB | **227662_at** | -561.26 | SYNPO2 |
| 229526_at | 832.70 | AQP11 | **223530_at** | 559.47 | TDRKH |
| 212661_x_at | 829.76 | LOC101060363 /// PPIA | **200960_x_at** | -557.13 | CLTA |
| 210786_s_at | -826.07 | FLI1 | **204720_s_at** | 557.03 | DNAJC6 |
| 242470_at | 822.24 | EID2B | **207026_s_at** | 555.69 | ATP2B3 |
| 226647_at | 820.99 | TMEM25 | **214260_at** | 540.70 | COPS8 |
| 201082_s_at | 819.92 | DCTN1 /// SLC4A5 | **201241_at** | 538.17 | DDX1 |
| 226470_at | 819.14 | GGT7 | **212987_at** | 537.46 | FBXO9 |
| 219983_at | 816.06 | HRASLS | **210617_at** | -537.41 | PHEX |
| 236440_at | 811.37 | NETO1 | **203745_at** | 532.74 | HCCS |
| 223550_s_at | 804.16 | CA10 | **51158_at** | 532.27 | FAM174B |
| 224888_at | 803.10 | EPT1 | **218720_x_at** | 531.02 | SEZ6L2 |
| 218300_at | -801.82 | PAGR1 | **223213_s_at** | -529.76 | ZHX1 |
| 201972_at | 800.89 | ATP6V1A | **225060_at** | 527.67 | LRP11 |
| 35776_at | -800.73 | ITSN1 | **236207_at** | -526.84 | SSFA2 |
| 231869_at | 798.79 | KIAA1586 | **207153_s_at** | -520.27 | GLMN |
| 238719_at | 797.23 | PPP2CA | **208752_x_at** | -513.85 | NAP1L1 |
| 209003_at | 794.47 | SLC25A11 | **213293_s_at** | -511.04 | TRIM22 |
| 206369_s_at | -793.58 | PIK3CG | **210786_s_at** | -508.97 | FLI1 |
| 635_s_at | 784.02 | PPP2R5B | **224378_x_at** | 508.66 | MAP1LC3A |
| 211615_s_at | 783.23 | LRPPRC | **227176_at** | 505.87 | SLC2A13 |
| 201293_x_at | 780.91 | LOC101060363 /// PPIA | **203610_s_at** | -503.75 | TRIM38 |
| 202016_at | 774.23 | MEST | **1555801_s_at** | 494.82 | ZNF385B |
| 208017_s_at | 770.15 | MCF2 | **211825_s_at** | -492.12 | FLI1 |
| 221805_at | 767.24 | NEFL | **223550_s_at** | 491.96 | CA10 |
| 201431_s_at | -760.24 | DPYSL3 | **244739_at** | -487.18 | RDX |
| 242876_at | -755.20 | AKT3 | **230773_at** | -485.19 | ZNF385D |
| 236638_at | 752.02 | AMER3 | **213270_at** | -484.27 | MPP2 |
| 202712_s_at | 751.50 | CKMT1A /// CKMT1B | **219894_at** | -482.52 | MAGEL2 |
| 223461_at | 751.14 | TBC1D7 | **204620_s_at** | -482.51 | VCAN |
| 208969_at | 751.02 | NDUFA9 | **235664_at** | -478.50 | --- |
| 212291_at | -750.39 | HIPK1 | **218760_at** | -474.47 | COQ6 |
| 206343_s_at | 747.67 | NRG1 | **204117_at** | -469.24 | PREP |
| 209297_at | -746.68 | ITSN1 | **207636_at** | -469.07 | SERPINI2 |
| 207153_s_at | 746.08 | GLMN | **202506_at** | -466.72 | SSFA2 |
| 223213_s_at | -743.12 | ZHX1 | **212072_s_at** | -459.73 | CSNK2A1 |
| 201757_at | 742.91 | NDUFS5 /// RPL10 | **200613_at** | 453.70 | AP2M1 |
| 207507_s_at | 741.64 | ATP5G3 | **222834_s_at** | -453.65 | GNG12 |
| 202698_x_at | 739.89 | COX4I1 | **213884_s_at** | 453.37 | TRIM3 |
| 205012_s_at | 733.68 | HAGH | **243317_at** | 450.14 | AX746627 |
| 229300_at | 731.05 | RAB3C | **219907_at** | -446.89 | FRS3 |
| 208826_x_at | 721.28 | HINT1 | **213333_at** | 445.75 | MDH2 |
| 218901_at | -717.59 | PLSCR4 | **220334_at** | 444.69 | RGS17 |
| 243998_at | 714.13 | KRT222 | **233337_s_at** | 439.93 | SEZ6L2 |
| 1553211_at | -710.81 | ANKFN1 | **224970_at** | -433.10 | NFIA |
| 214157_at | 710.74 | GNAS | **1554133_at** | -426.98 | RUFY2 |
| 213149_at | 708.62 | DLAT | **1556159_at** | 426.67 | --- |
| 220794_at | 703.41 | GREM2 | **223011_s_at** | 426.61 | OCIAD1 |
| 222125_s_at | 703.41 | P4HTM | **235852_at** | -424.62 | STON2 |
| 228062_at | 702.88 | NAP1L5 | **226259_at** | 395.37 | EXOC6 |
| 243317_at | 702.57 | AX746627 | **228262_at** | -393.10 | MAP7D2 |
| 209545_s_at | -700.93 | RIPK2 | **214078_at** | -392.51 | AF070581 |
| 208846_s_at | 699.22 | VDAC3 | **223174_at** | 386.92 | BTBD10 |
| 206404_at | 698.44 | FGF9 | **229526_at** | 385.79 | AQP11 |
| 203339_at | 692.58 | SLC25A12 | **235656_s_at** | 378.63 | --- |
| 216056_at | -682.01 | CD44 | **231120_x_at** | 376.74 | PKIB |
| 238658_at | 679.47 | --- | **219271_at** | -373.37 | GALNT14 |
| 229135_at | 678.32 | FASTKD2 | **232377_at** | 369.63 | NXPH1 |
| 219301_s_at | 677.36 | CNTNAP2 | **211493_x_at** | -363.84 | DTNA |
| 229649_at | 674.64 | NRXN3 | **207501_s_at** | 358.27 | FGF12 |
| 200978_at | 671.97 | MDH1 | **226813_at** | -357.80 | NTPCR |
| 203069_at | 670.36 | SV2A | **49077_at** | -357.30 | PPME1 |
| 221880_s_at | 668.30 | FAM174B | **1555313_a_at** | -357.12 | MCF2 |
| 210617_at | -665.55 | PHEX | **242470_at** | 355.57 | EID2B |
| 204749_at | 664.56 | NAP1L3 | **211962_s_at** | -352.94 | ZFP36L1 |
| 213938_at | 659.83 | ERC2 | **210108_at** | 351.43 | CACNA1D |
| 225060_at | 658.04 | LRP11 | **233323_at** | 346.10 | --- |
| 226653_at | 657.32 | MARK1 | **236638_at** | 343.31 | AMER3 |
| 229097_at | -655.31 | DIAPH3 | **244647_at** | 341.20 | WBP11 |
| 218597_s_at | 653.25 | CISD1 | **232195_at** | 337.05 | GPR158 |
| 212425_at | 647.89 | SCAMP1 | **227226_at** | -327.14 | MRAP2 |
| 228262_at | 641.12 | MAP7D2 | **231967_at** | -324.54 | PHF20L1 |
| 226259_at | 632.72 | EXOC6 | **202779_s_at** | -323.25 | UBE2S |
| 226339_at | 627.11 | TRUB1 | **209186_at** | 321.53 | ATP2A2 |
| 1554835_a_at | -620.10 | B3GNT5 | **202825_at** | 319.45 | SLC25A4 |
| 223011_s_at | 619.31 | OCIAD1 | **202133_at** | -318.98 | WWTR1 |
| 207508_at | 615.03 | ATP5G3 | **201757_at** | 318.76 | NDUFS5 /// RPL10 |
| 219896_at | 611.79 | CALY | **207984_s_at** | -317.72 | MPP2 |
| 228835_at | 611.61 | RP4-758J24.5 | **211072_x_at** | 312.19 | TUBA1B |
| 208898_at | 603.55 | ATP6V1D | **210736_x_at** | -308.84 | DTNA |
| 205795_at | 601.23 | NRXN3 | **238673_at** | 302.97 | SAMD12 |
| 204141_at | 600.64 | TUBB2A | **223518_at** | 302.74 | DFFA |
| 204001_at | 595.44 | SNAPC3 | **37226_at** | 301.11 | BNIP1 |
| 200786_at | 595.14 | PSMB7 | **220794_at** | -298.66 | GREM2 |
| 240942_at | 591.26 | MPHOSPH8 | **206408_at** | -297.62 | LRRTM2 |
| 221847_at | 589.81 | LOC100129361 | **203094_at** | 296.16 | MAD2L1BP |
| 226649_at | 589.18 | PANK1 | **208845_at** | 290.53 | VDAC3 |
| 204933_s_at | -586.93 | TNFRSF11B | **223532_at** | 287.24 | ANKRD39 |
| 213921_at | 576.97 | SST | **222099_s_at** | -286.31 | LSM14A |
| 225969_at | 576.69 | ALKBH6 | **241801_at** | 286.25 | PGAP1 |
| 242317_at | 574.75 | HIGD1A | **205531_s_at** | 284.34 | GLS2 |
| 203889_at | 574.08 | SCG5 | **218706_s_at** | -279.26 | GRAMD3 |
| 209242_at | 563.93 | PEG3 | **208457_at** | 274.33 | GABRD |
| 209694_at | 561.76 | PTS | **221847_at** | 270.24 | LOC100129361 |
| 213278_at | 558.39 | MTMR9 | **226649_at** | -264.62 | PANK1 |
| 203517_at | 551.28 | MTX2 | **222513_s_at** | -262.96 | SORBS1 |
| 202825_at | 550.06 | SLC25A4 | **219688_at** | 258.30 | BBS7 |
| 213558_at | 546.21 | PCLO | **244688_at** | 255.28 | --- |
| 238569_at | -545.85 | GABBR1 | **213793_s_at** | -252.90 | HOMER1 |
| 232791_at | -540.04 | --- | **214434_at** | 246.97 | HSPA12A |
| 238673_at | 537.41 | SAMD12 | **205646_s_at** | -243.70 | PAX6 |
| 231120_x_at | 536.58 | PKIB | **217942_at** | 235.64 | MRPS35 |
| 208870_x_at | 535.46 | ATP5C1 | **220889_s_at** | 234.75 | CA10 |
| 203999_at | 529.01 | SYT1 | **229097_at** | -234.26 | DIAPH3 |
| 218163_at | 524.82 | MCTS1 | **209570_s_at** | 229.91 | NSG1 |
| 225719_s_at | 519.37 | MRPL55 | **230498_at** | 226.43 | MCHR1 |
| 223604_at | 519.00 | GARNL3 | **207772_s_at** | 221.75 | PRMT8 |
| 244099_at | 517.14 | CACNG2 | **217976_s_at** | 219.64 | DYNC1LI1 |
| 201322_at | 514.50 | ATP5B | **37950_at** | -217.14 | PREP |
| 216903_s_at | 514.48 | MICU1 | **225969_at** | -210.76 | ALKBH6 |
| 218969_at | 506.84 | PAM16 | **1552736_a_at** | 210.32 | NETO1 |
| 1552736_a_at | 505.24 | NETO1 | **238889_at** | 208.86 | AGBL5 |
| 241399_at | 498.01 | FAM19A2 | **204933_s_at** | 202.41 | TNFRSF11B |
| 223367_at | 496.71 | DNAJC30 | **213217_at** | -197.88 | ADCY2 |
| 223239_at | 493.55 | GSKIP | **235253_at** | -189.98 | RAD1 |
| 204721_s_at | 492.02 | DNAJC6 | **201753_s_at** | 188.65 | ADD3 |
| 1556151_at | 481.57 | ITFG1 | **217841_s_at** | -187.54 | PPME1 |
| 37226_at | 475.91 | BNIP1 | **226822_at** | -186.99 | STOX2 |
| 218568_at | 472.45 | AGK | **240111_at** | -186.16 | RHOBTB3 |
| 214665_s_at | 466.90 | CHP1 | **211685_s_at** | -185.50 | NCALD |
| 203396_at | 465.34 | PSMA4 | **207842_s_at** | -183.70 | CASC3 /// MIR6866 |
| 236838_at | 464.22 | SRCIN1 | **218300_at** | 179.25 | PAGR1 |
| 217782_s_at | 464.06 | GPS1 | **213486_at** | 178.30 | COPG2IT1 |
| 213720_s_at | 463.75 | SMARCA4 | **231763_at** | 175.80 | POLR3A |
| 230137_at | 457.39 | TMEM155 | **213720_s_at** | 175.71 | SMARCA4 |
| 239265_at | 451.10 | SLC35G1 | **208999_at** | -163.82 | SEPT8 |
| 226767_s_at | 445.67 | FAHD1 | **1554524_a_at** | -161.56 | OLFM3 |
| 212686_at | 438.35 | PPM1H | **208430_s_at** | -153.98 | DTNA |
| 208752_x_at | -436.27 | NAP1L1 | **205230_at** | -153.34 | RPH3A |
| 207984_s_at | 432.46 | MPP2 | **214436_at** | -148.53 | FBXL2 |
| 219907_at | 428.90 | FRS3 | **1557286_at** | -144.32 | --- |
| 213366_x_at | 418.93 | ATP5C1 | **230137_at** | -144.25 | TMEM155 |
| 218671_s_at | 416.88 | ATPIF1 | **233910_at** | -142.07 | TMEFF2 |
| 215440_s_at | 407.31 | BEX4 | **212425_at** | 141.58 | SCAMP1 |
| 206408_at | 406.62 | LRRTM2 | **225779_at** | -139.92 | SLC27A4 |
| 225485_at | 401.23 | CEP41 | **204514_at** | 137.69 | DPH2 |
| 238504_at | 396.51 | C6orf57 | **222125_s_at** | -134.63 | P4HTM |
| 1559257_a_at | 393.65 | MAGI1 | **209796_s_at** | -131.07 | CNPY2 |
| 228109_at | 385.75 | RASGRF2 | **218969_at** | -127.54 | PAM16 |
| 219271_at | 381.32 | GALNT14 | **229649_at** | -126.30 | NRXN3 |
| 238115_at | 380.56 | DNAJC18 | **218087_s_at** | 118.86 | SORBS1 |
| 201410_at | 377.71 | PLEKHB2 | **208751_at** | 117.27 | NAPA |
| 209444_at | 377.47 | RAP1GDS1 | **214170_x_at** | -112.87 | FH |
| 210650_s_at | 374.17 | PCLO | **219659_at** | -111.40 | ATP8A2 |
| 202961_s_at | 366.79 | ARMC2-AS1 /// ATP5J2 | **227455_at** | 111.28 | C6orf136 |
| 203020_at | 363.15 | RABGAP1L | **1554592_a_at** | 111.03 | SLC1A6 |
| 214306_at | 356.44 | OPA1 | **204721_s_at** | 104.67 | DNAJC6 |
| 204514_at | 354.13 | DPH2 | **227219_x_at** | 98.01 | MAP1LC3A |
| 203862_s_at | 352.47 | ACTN2 | **206046_at** | 95.42 | ADAM23 |
| 218477_at | 340.71 | TMEM14A | **201431_s_at** | 94.72 | DPYSL3 |
| 208678_at | 335.38 | ATP6V1E1 | **221880_s_at** | -93.62 | FAM174B |
| 226154_at | 312.59 | DNM1L | **231935_at** | 92.22 | ARPP21 |
| 244739_at | 302.38 | RDX | **226752_at** | -89.86 | FAM174A |
| 209935_at | 300.35 | ATP2C1 | **204365_s_at** | -85.40 | REEP1 |
| 226822_at | 291.48 | STOX2 | **224869_s_at** | 84.45 | MRPS25 |
| 238466_at | 284.75 | --- | **1556151_at** | 84.07 | ITFG1 |
| 211072_x_at | 280.46 | TUBA1B | **1555800_at** | 76.31 | ZNF385B |
| 201873_s_at | -267.17 | ABCE1 | **216903_s_at** | 73.47 | MICU1 |
| 231967_at | 263.51 | PHF20L1 | **229925_at** | -71.90 | SLC6A17 |
| 226813_at | -251.62 | NTPCR | **244099_at** | 66.20 | CACNG2 |
| 223518_at | 250.71 | DFFA | **213149_at** | 64.88 | DLAT |
| 225535_s_at | 239.31 | TIMM23 /// TIMM23B | **224913_s_at** | -61.54 | TIMM50 |
| 226752_at | 239.07 | FAM174A | **203862_s_at** | 61.13 | ACTN2 |
| 209902_at | 216.63 | ATR | **239935_at** | 59.94 | MDGA2 |
| 238871_at | 198.33 | MLLT4 | **219752_at** | -51.13 | RASAL1 |
| 1554133_at | -181.61 | RUFY2 | **244463_at** | 46.62 | ADAM23 |
| 218760_at | -177.18 | COQ6 | **227401_at** | 41.56 | IL17D |
| 217942_at | 174.33 | MRPS35 | **223461_at** | 35.45 | TBC1D7 |
| 209589_s_at | -143.68 | EPHB2 | **219660_s_at** | 34.85 | ATP8A2 |
| 208845_at | 142.16 | VDAC3 | **225719_s_at** | -34.74 | MRPL55 |
| 209796_s_at | 126.04 | CNPY2 | **236277_at** | 24.56 | AF070581 |
| 219752_at | 89.03 | RASAL1 | **209902_at** | 23.57 | ATR |
| 213270_at | -87.46 | MPP2 | **635_s_at** | 23.03 | PPP2R5B |
| 214170_x_at | 66.19 | FH | **215021_s_at** | 20.07 | NRXN3 |
| 241758_at | 44.32 | NUP93 | **208451_s_at** | -16.22 | C4A /// C4B /// C4B_2 |
| 204720_s_at | -10.99 | DNAJC6 | **213808_at** | 13.17 | ADAM23 |
| 212072_s_at | 10.95 | CSNK2A1 | **218901_at** | -10.09 | PLSCR4 |
| 203724_s_at | -6.01 | RUFY3 | **206343_s_at** | -3.26 | NRG1 |
| 1557820_at | 0.02 | AFG3L2 | **238462_at** | -3.05 | UBASH3B |
