# Supplementary figures and images for: An Artificial Neural Network Integrated Pipeline for Biomarker Discovery Using Alzheimer's Disease as a Case Study
Source: Comput Struct Biotechnol J. 2018 Feb 21;16:77–87. doi: 10.1016/j.csbj.2018.02.001 (PMC6026215; doi:10.1016/j.csbj.2018.02.001)

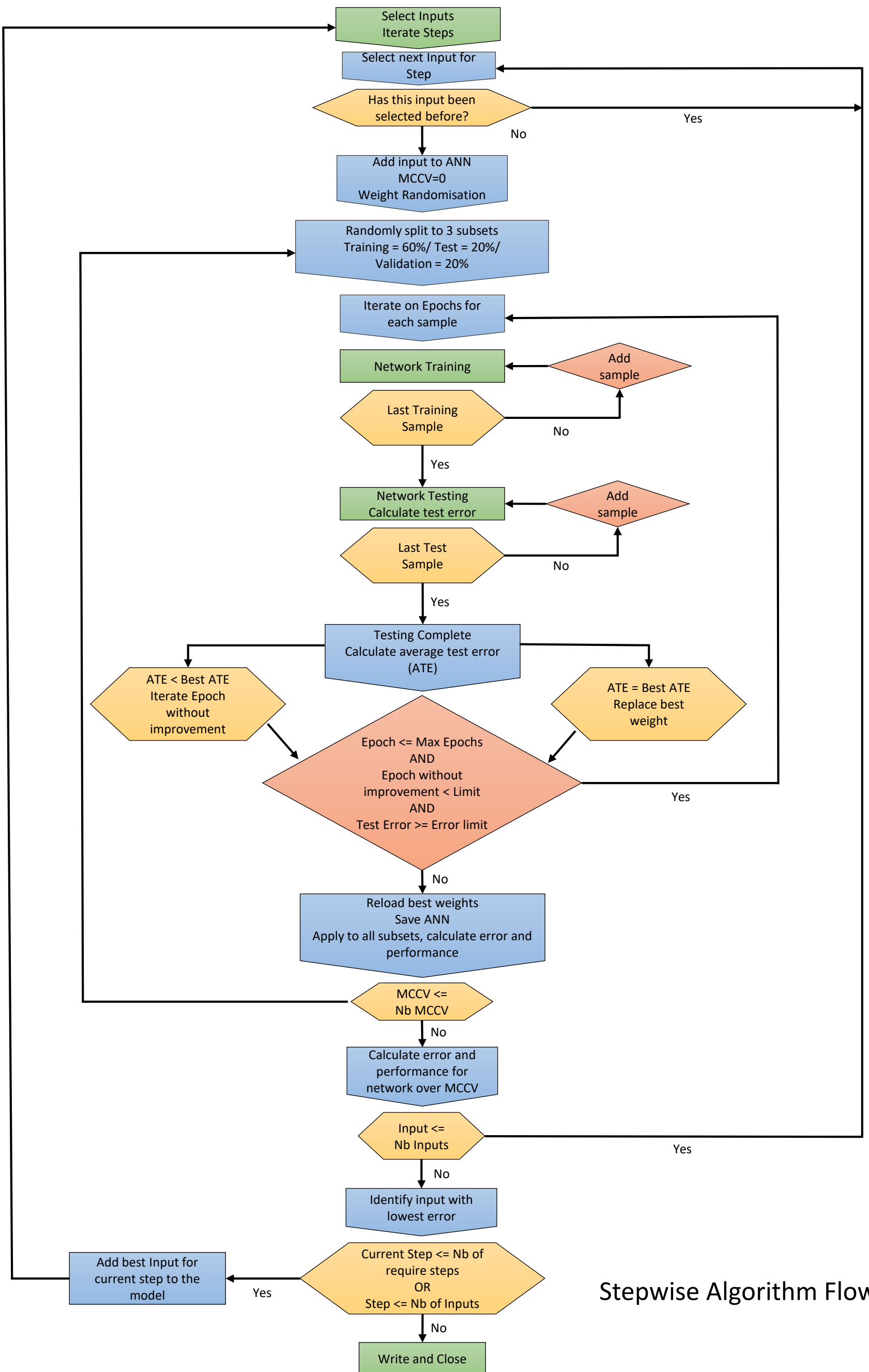

Stepwise Algorithm Flowchart

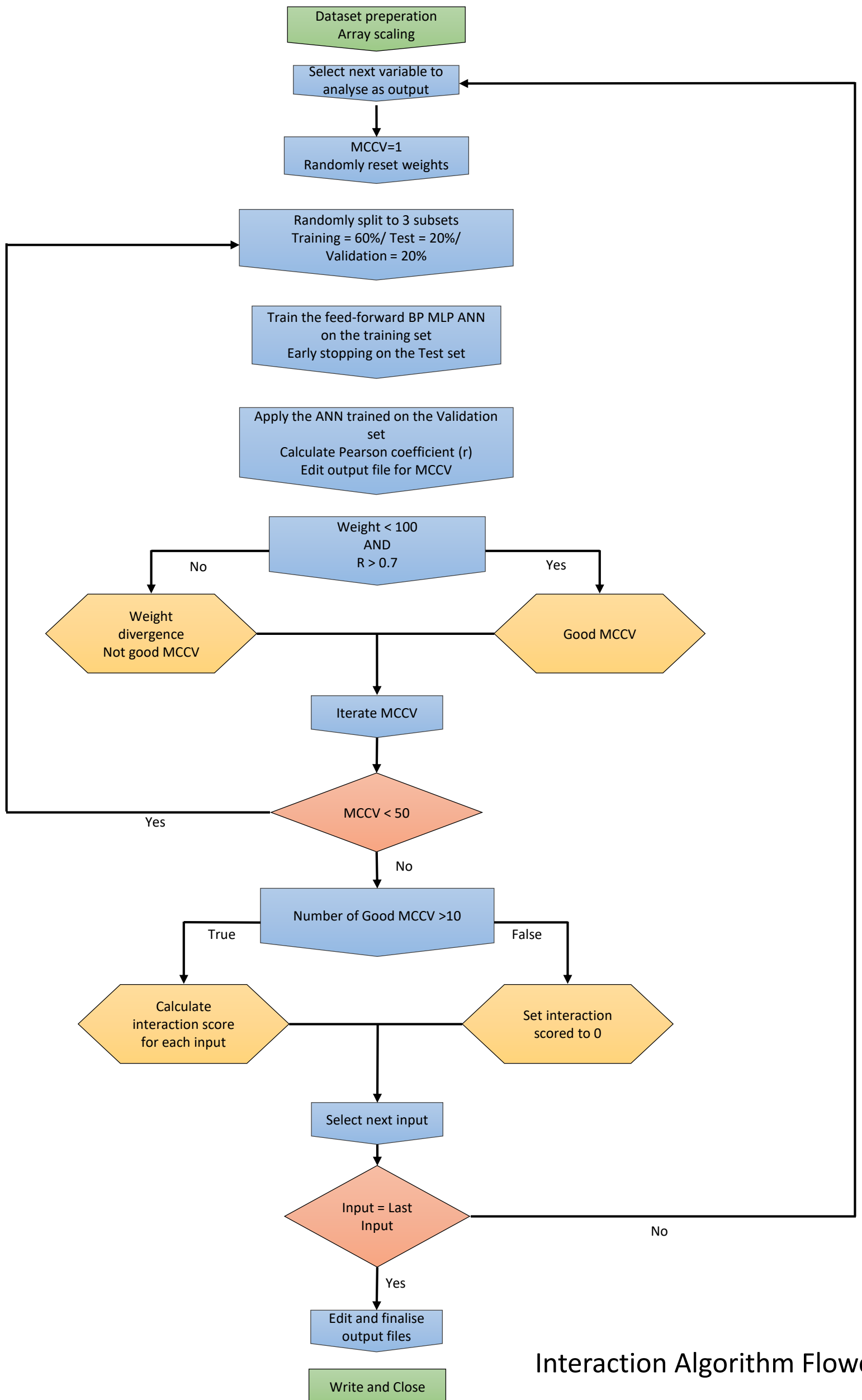

Interaction Algorithm Flowchart

Supplement: Supplementary file 3 — Stepwise and Interaction Algorithm Flowcharts. [file mmc3.pdf]
